# Preliminary Study on Immediate Postoperative CT Images and Values of the Modular Polyetheretherketone Based Total Knee Arthroplasty: An Observational First-in-Human Trial

**DOI:** 10.3389/fsurg.2022.809699

**Published:** 2022-02-14

**Authors:** Zhengyu Cai, Xinhua Qu, Yaochao Zhao, Zhiguo Yuan, Liangjun Zheng, Teng Long, Qiuying Yao, Bing Yue, You Wang

**Affiliations:** ^1^Department of Bone and Joint Surgery, Renji Hospital, School of Medicine, Shanghai Jiao Tong University, Shanghai, China; ^2^Department of Orthopedics, Renji Hospital, School of Medicine, Shanghai Jiao Tong University, Shanghai, China; ^3^Department of Radiology, Renji Hospital, School of Medicine, Shanghai Jiao Tong University, Shanghai, China

**Keywords:** arthroplasty, polyetheretherketone, computed tomography, bone density, prosthetic overhang

## Abstract

**Background:**

Total knee arthroplasty (TKA) is now frequently performed and is highly successful. However, patient satisfaction after TKA is often difficult to achieve. Because of the presence of metallic prosthetic knee joints, there is a lack of imaging tools that can accurately assess the patient's postoperative prosthetic position, soft tissue impingement, and periprosthetic bone density after TKA. We conducted a clinical trial of the world's first totally modular polyetheretherketone (PEEK) TKA and determined the bone density values in the stress concentration area around the prosthesis based on postoperative computed tomography data to reconstruct a three-dimensional model of the PEEK prosthetic knee joint after implantation. Based on the model, the overhang of the prosthesis was measured at various locations on the prosthesis.

**Methods:**

All patients who underwent PEEK-based TKA were postoperatively assessed with radiography and computed tomography (CT). Hounsfield units (HUs) for the different components of the quantitative CT assessment were measured separately.

**Results:**

Ten patients (nine female and one male) aged 59–74 (mean 66.9, median 67) years were included. The HU values were as follows: PEEK prosthesis mean 182.95, standard deviation (SD) 4.90, coefficient of variation (CV) 2.68; polyethylene mean −89.41, SD 4.14, CV −4.63; lateral femoral osteochondral mean 192.19, SD 55.05, CV 28.64; lateral tibial osteochondral mean 122.94, SD 62.14, CV 42.86; medial femoral osteophyte mean 180.76, SD 43.48, CV 24.05; and medial tibial osteophyte mean 282.59, SD 69.28, CV 24.52. Analysis of the data at 1, 3, and 6 months showed that the mean PE (*p* = 0.598) and PEEK (*p* = 0.916) measurements did not change with the time of measurement. There was a decrease in bone mineral density in the lateral tibia at 3 months (*p* = 0.044). Otherwise, there was no significant change in bone density in other regions (*p* = 0.124–0.803). There was no overhang in all femoral prostheses, whereas there were two cases of overhang in tibial prostheses. Overhang measurements do not differ significantly across time points. The overhang measurements were not significantly different at all time points (*p* = 0.186–0.967).

**Conclusion:**

PEEK knee joint prosthesis has excellent CT compatibility. The change in periprosthetic bone volume during the follow-up period can be determined using the HU value after CT scan, while the prosthesis position can be assessed. This assessment may potentially guide future improvements in knee prosthesis alignment techniques and artificial knee prosthesis designs.

## Introduction

Total knee arthroplasty (TKA) is one of the most successful orthopedic procedures for patients with end-stage arthritis, offering the opportunity to restore joint motion and improve the quality of life of older patients with knee osteoarthritis, severe rheumatoid arthritis, and tumors (1, 2). Traditional implants for total knee replacement primarily comprise titanium or cobalt-chromium alloys, forming the femoral and tibial components (1). Despite the decades-long history of success with metallic prostheses, there are still material defects that require improvement. For example, adverse reactions due to metal allergy have been reported after TKA in some patients (3–5).

The prevalence of contact allergy to nickel, cobalt and chromium in the population has been estimated at 13% (20% in women), 2.4%, and 1.1%, respectively (6–8). Eben et al. suggested that complications related to metal allergy are often underestimated, which reaches 29.6% (7). Complications associated with allergy to metal implants include dermatitis, poor wound healing, infection-like reactions, oozing, pain, and loosening (9). Although the mechanism by which metal allergy leads to TKA failure is unclear (10), there is consensus among surgeons to avoid postoperative discomfort, such as joint swelling, skin pruritus, and decreased range of motion (ROM), due to metal allergies.

Conversely, the stress-masking effect of the long-term fixation of metal prostheses cannot be avoided with bone cement fixation, which theoretically leads to the loss of bone volume around the prosthesis, resulting in periprosthetic bone resorption, pathological fractures, and loosening (2). The PEEK implant has a significantly lower stress-shielding effect compared to metal and the strain after implantation is not significantly different compared to intact bone (11). Bone loss due to metal implants is also considered a common side effect of frequent knee replacements (12–14). According to Wolff's law, this would be of great significance in avoiding loss of bone around the prosthesis. The imaging assessment of postoperative periprosthetic infection and aseptic loosening is hindered by the large metallic artifacts in imaging tests such as computed tomography (CT) and magnetic resonance imaging, which greatly limits the early diagnosis of infection and loosening after TKA. It is also difficult to accurately analyze the match of the prosthesis-osteointegration interface, prosthesis boundary, and osteotomy surface boundary after metal prosthesis placement, which may affect the development of high-performance artificial knee joint replacement technology.

Polyetheretherketone (PEEK), which has an elastic modulus more similar to bone than to metals (15, 16), better biocompatibility, and enables osteogenesis around the implant (17), has been used in intervertebral lumbar cages, screws, and cranial patches in orthopedic surgery (18). Moreover, research on PEEK as an artificial knee material has also progressed (19). A clinical trial of PEEK afemoral components and all-polyethylene tibial components has been reported recently (20), but there has been no clinical study about the totally modular PEEK knee joint prosthesis until now.

Our previous animal experiments demonstrated that cemented PEEK-on-highly cross-linked polyethylene (HXLPE) artificial knee joints have good biosafety in goat models (21). PEEK weight-bearing surfaces have better prevention of periprosthetic and contralateral cartilage degeneration than CoCrMo (cobalt-chromium-molybdenum alloy, vitallium) weight-bearing surface microprostheses (22). Bone density examination after PEEK artificial joint implantation showed an early periprosthetic femoral transient mild decrease, whereas no change was observed around the tibial prosthesis (21).

In 2016, Suzhou SinoMed Biomaterials Co., Ltd., China, in collaboration with Solvay, USA, developed the PEEK Knee system. Based on the positive results of the overall bio-completeness, *in-vitro* mechanical strength, and simulated wear experiments of the PEEK Knee system, we provided the PEEK artificial knee joint to 10 patients after obtaining ethical approval from the Shanghai Jiao Tong University School of Medicine, Renji Hospital Ethics Committee (KY2021-025). We performed X-ray, CT, and measurement studies on early prosthesis position, peripheral bone density, cement fixation status, and prosthesis-bone border matching after PEEK implantation to provide reliable methods and basic data for further quantitative imaging analyses for postoperative follow-up.

## Materials and Methods

The current trial was registered at ClinicalTrials.gov (NCT04927104) in June 2021. We conducted a one-arm parallel clinical exploratory study in adult patients undergoing PEEK TKA from June 2021 to July 2021 at a single center. Written consent for participation in the study was obtained from each patient.

### Study Patients

All patients meeting the inclusion criteria were assessed for eligibility by three trained study coordinators. The inclusion criteria were as follows: (1) those aged 55–74 years; (2) patients who were skeletally mature; (3) patients with indications for TKA; and (4) patients whose diseased knee had not undergone TKA surgery. The exclusion criteria were as follows: (1) neuromuscular insufficiency that might have led to postoperative knee instability or gait abnormalities; (2) patients with bilateral knee disease who were expected to require bilateral knee replacement during the study (i.e., within the following 12 months); (3) alcohol or substance abusers; (4) those with a body mass index (BMI) of >35 kg/m^2^; (5) patients with severe diabetes mellitus (fasting glucose > 10 mmol/L); (6) pregnant or lactating women; and (7) those with other comorbidities that would have limited their participation in the study, prevented compliance with follow-up, or affected the scientific validity and integrity of the study. Ten patients were included in the first trial, in accordance with the inclusion criteria and clinical study design.

The 10 patients had a mean age of 66.9 (59–74) years, median age of 67 years, mean weight of 62.4 (53–89) kg, median weight of 60 kg, mean height of 158.4 cm, median height of 158.5 (148–175) cm, mean BMI of 24.87 (17.31–33.50) kg/m^2^, and median BMI of 24.72 kg/m^2^ (Supplementary Table 1).

### Manufacturing the PEEK Prosthesis

**Figure 1:**
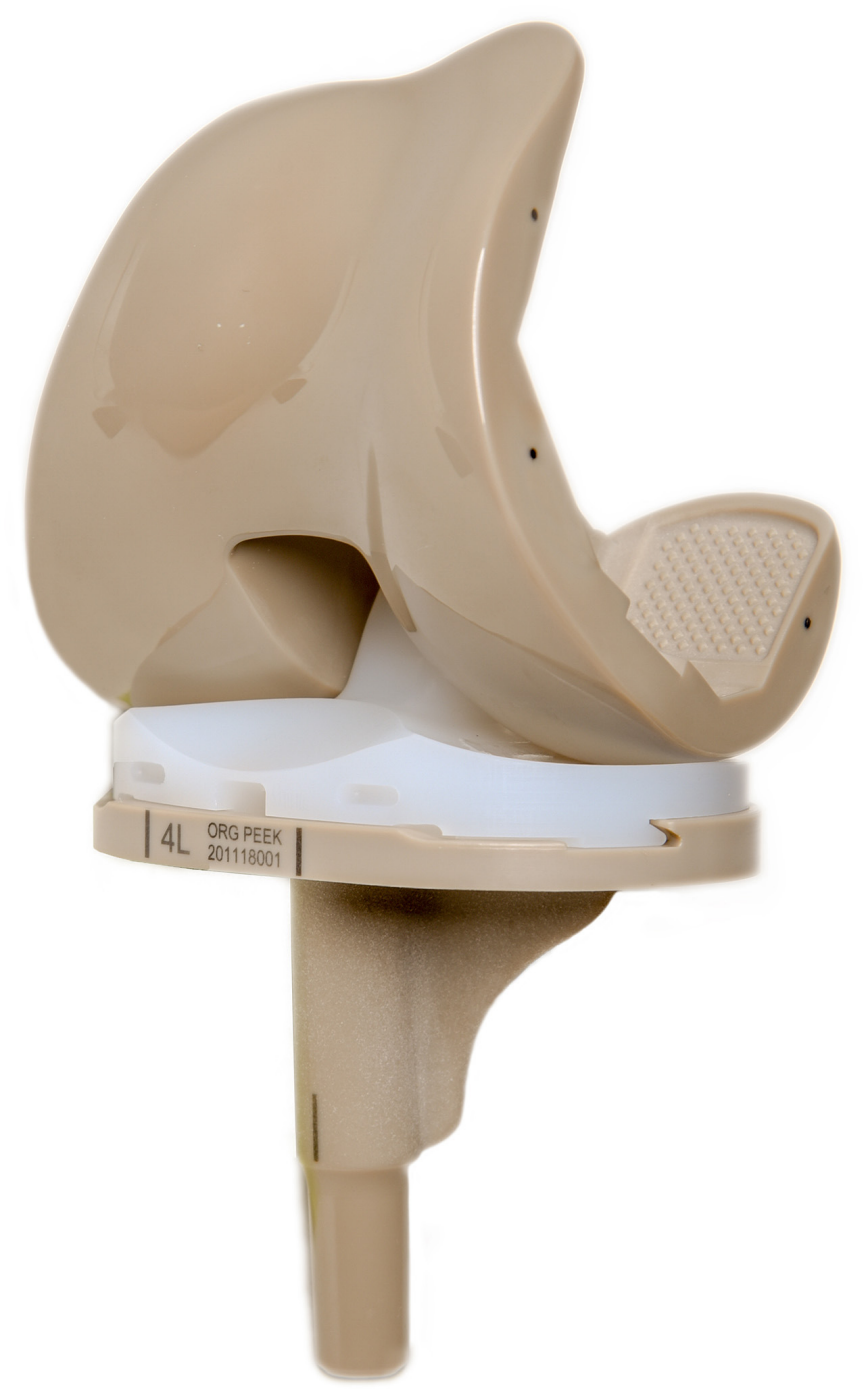
The complete knee prosthesis consists of the PEEK femoral component, PEEK tibial component, and an all-polyethylene bearing. PEEK, polyetheretherketone.

The PEEK artificial knee joint consists of four parts: the femoral condyle (PEEK), tibial tray (PEEK), bearing (HXLPE), and patellar component (HXLPE). The femoral condyle and tibial tray were injection-molded from Solvay PEEK pellets and irradiated for sterilization. The bearing and patellar component were processed from Quadrant highly cross-linked polyethylene material and sterilized using ethylene oxide. The PEEK components and HXLPE bearing (Zeniva PEEK ZA-500, Chirulen HXLPE 1020X) were provided by Suzhou SinoMed Biomaterials Co. Ltd. (Figure 1).

### Surgical Procedures

All procedures were performed under general anesthesia by the senior author. The surgical technique was the same for all the knee joints. All procedures were performed using an ~15-cm-long anterior skin incision at the midline of the knee.

Every patella underwent surface replacement, which was essentially guaranteed to be similar in thickness to the preoperative patella. An intramedullary femoral guide and an extramedullary tibial guide were used to adjust the force lines. The size and rotation of the components and bearing were selected using a joint spacer and tensor, respectively. All components were cemented. During each procedure, the surgeon routinely measured the fit of the prosthesis relative to the osteotomy margin after bone removal. The intraoperative C-arm X-ray machine revealed a good prosthesis position.

### Radiographic Evaluation

Patients underwent preoperative radiography, CT, and X-ray postoperatively. The specific imaging systems used were the DigitalDiagnost (Philips, Amsterdam, Netherlands) for X-rays and uCT 780 (United Imaging Healthcare, Shanghai, China) and Optima CT680 series (GE Healthcare, Chicago, IL) for CT. We performed detailed imaging evaluations on 10 cases measured at 1 month, 10 cases at 3 months, and seven cases at 6 months.

**Figure 2:**
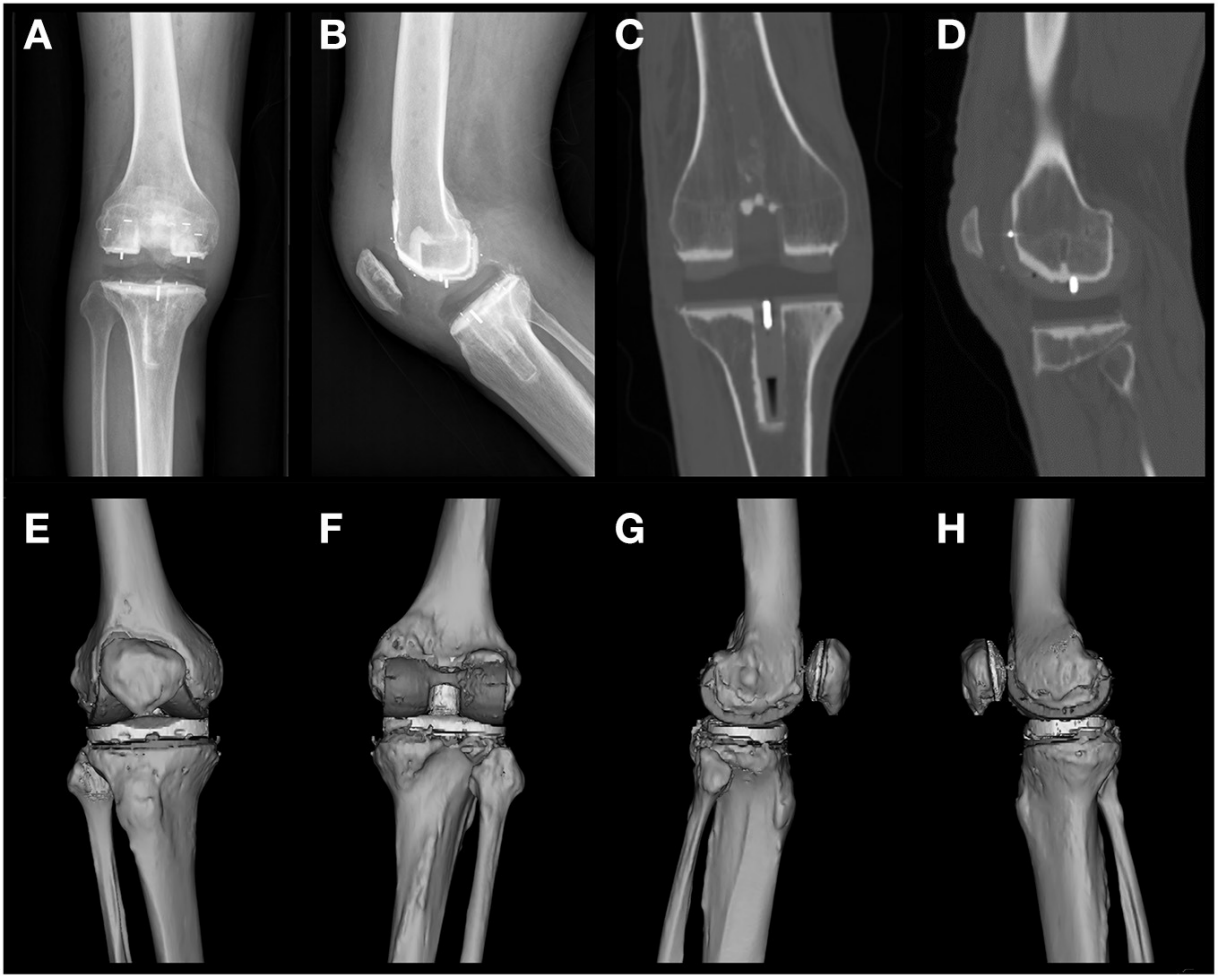
Postoperative X-rays of frontal and lateral views of patient 1 **(A,B)**, images of postoperative CT coronal intermediate and lateral condyle central layers **(C,D)**, and a preview of the 3D reconstructed model **(E–H)** are shown. CT level selection: coronal, sagittal, and axial positions consistent with the joint force lines. The most central metal line was selected according to the position of the central tibial plateau prosthesis and the central lateral femoral condyle. The most central position of the metal wire was selected as the intercept plane **(C)**. Mimics reconstruction software was used to arrange the viewing angles in the order of anterior, posterior, lateral, and medial positions **(E–H)**.

The three-dimensional (3D) model reconstruction was based on different window widths for different tissues according to the data obtained from CT observation by Mimics version 21.0.0.406 (Materialise, Leuven, Belgium). The specific parameters were as follows: PEEK material, 120–220 Hounsfield unit (HU); bone and bone cement, 220–2,000 HU; and HXLPE,−100-−60 HU. The sketch obtained was the 3D reconstruction model shown in Figure 2 and Supplementary Figures 1–9.

**Figure 3:**
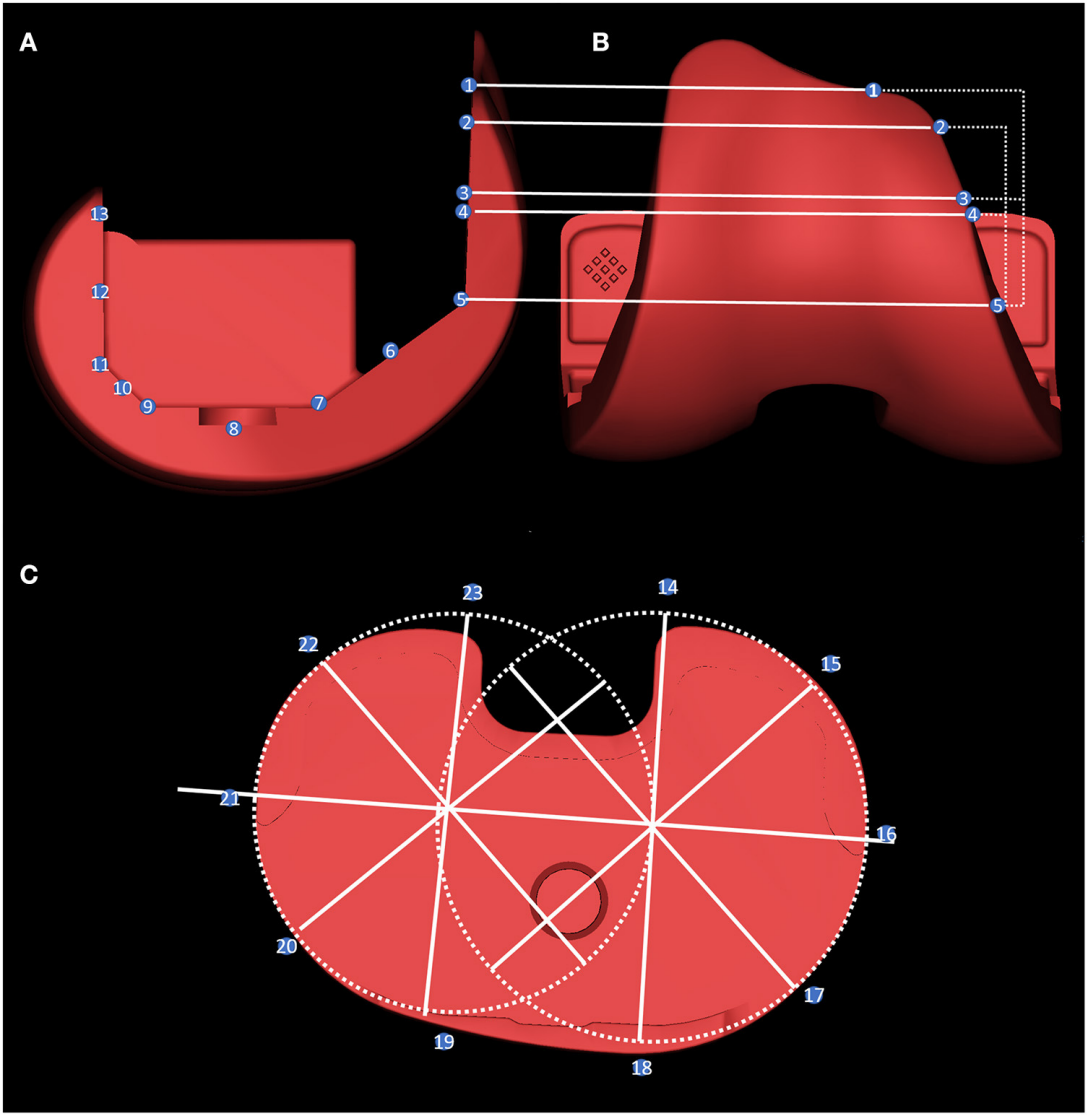
The overhang measurement method. The femoral side was measured by taking the five planes of the prosthesis, the midpoint of each plane, and the length of the prosthesis and the bone-implant bed on the horizontal line of the inner and outer edges of points 1–13 **(A,B)**. For tibial overhang measurement: two external tangential circles were made at the interior and lateral edge of the prosthesis, and the difference between the length of the prosthesis and the bone-implant bed was measured every 45° on these radii at points 14–23 **(C)**.

**Figure 4:**
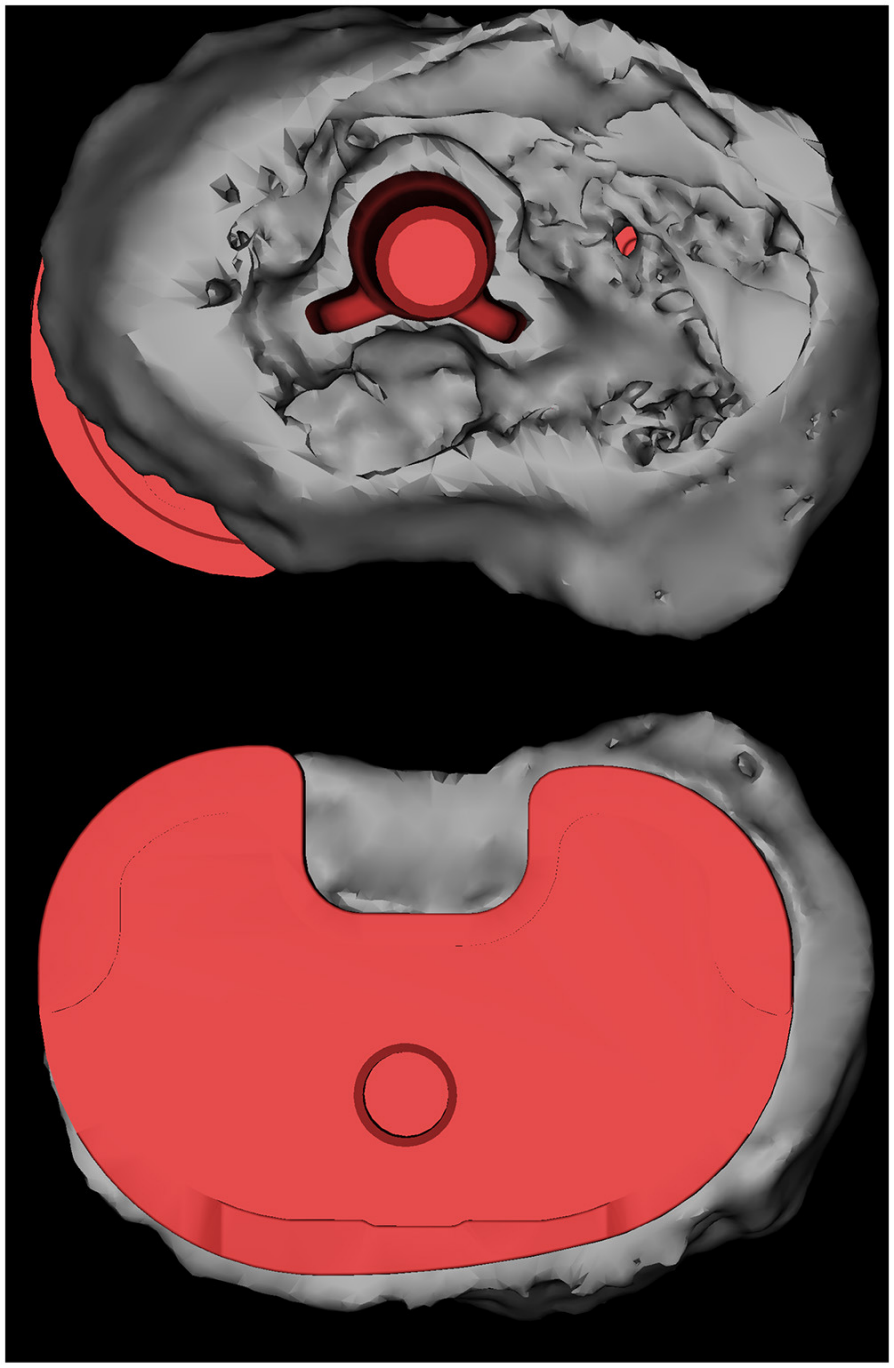
The results of the 3D reconstruction of a patient are shown, with the HXLPE bearing, patella and patellar prosthesis, femoral stem, and a part of the tibial stem and fibula removed. The prosthesis was superposed by the corresponding type of prosthesis, and the placement of the prosthesis was determined by matching the results of the previous 3D reconstruction. A tibial plateau prosthesis with a posteromedial overhang is shown. HXLPE, highly cross-linked polyethylene.

Tiny particles that are not reconstructed from bone tissue, metal and cement were defined as noise. The location of these noises is often outside the bone tissue and joint prosthesis, so their removal does not affect the measurement of the prosthesis position. The stereolithography file prosthesis models obtained from the manufacturer were directly matched and superposed for the assembly of the models used in Figures 3, 4, in a manner similar to that previously reported (23).

**Figure 5:**
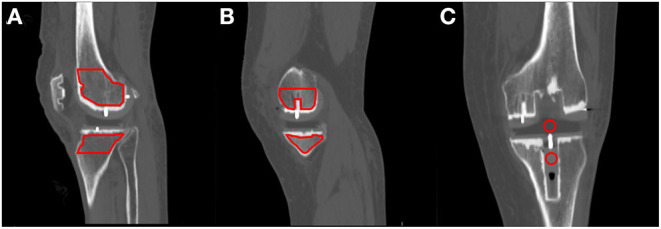
The CT density measurement method. In the sagittal position, the lateral and medial condyles were selected centrally (midpoint of the metal alignment line), and the cancellous area was encircled using the Weasis polygon tool **(A,B)**. The HU values of the HXLPE bearing and the PEEK tibial prosthesis stem (stem) were selected in the coronal position **(C)**. The ROI is distributed up to the highest area of the femoral prosthesis to the joint line a, and the lowest area of the tibial plateau is visible on CT to the joint line. CT, computed tomography; HU, Hounsfield unit; HXLPE, highly cross-linked polyethylene; PEEK, polyetheretherketone; ROI, region-of-interest.

The methods for measuring the HU values of the PEEK prosthetic stem, HXLPE bearing, and osteophyte are shown in Figure 5. We measured density data at 1, 3, and 6 months. Weasis Medical Viewer was used to measure the HU values of the HXLPE bearing and the PEEK tibial prosthetic stem in a postoperative CT coronal view of the selected area. The region-of-interest (ROI) was encircled using the circle tool while ensuring that no non-tissue of interest was involved, and the software automatically generated the average HU values in the area.

According to the results of previous pre-experiments (21), the HU values of these two areas are relatively stable and not easily disturbed by metallic artifacts or bone cement. The lateral and medial condyles were selected in the sagittal superior sagittal position (the midpoint of the metal positioning line), and the osteophyte area of the polygon tool was used to circle the area from the highest to the joint line of the femoral prosthesis and the area from the lowest to the joint line visible on the medial tibial plateau. The software automatically generated the HU values within the area.

The method of measuring the overhang of each component of the prosthesis is shown in Figure 3. Before measurement, the prosthesis within the reconstructed model was replaced with an engineered prosthesis model to obtain more accurate edge measurements. Figure 4 shows a schematic diagram of the femoral and tibial overhang measurement methods. The overhang was measured on the femoral side by taking the five plane turning points of the prosthesis, the midpoint of each plane, and the position of the inner and outer edges of the upper edge of the prosthesis.

The length of the prosthesis and bone-implant bed on the horizontal line was measured on the condyles of the femur, where points 1, 2, 5, 7, 9, 11, and 13 were the locations of the turning points of the union surfaces of each facet prosthesis (Figure 3A). Moreover, points 3, 4, 6, 8, 10, and 12 were the locations of the midpoints between each turning point of the prosthesis on the anterior femoral condyles. Point 3 was the midpoint of points 1 and 5, while point 4 was the midpoint of points 2 and 5.

When measuring the lateral tibial overhang, the circles tangential to the lateral edges of the two platforms were drawn on the medial and lateral platforms. The centers of the two circles were connected as the transverse axis of the prosthesis. Moreover, a radius was drawn at the center of the two circles at 45°, and the length of the prosthesis and the bone-implant bed was measured on these radii at points 14–23 (Figure 3B). The bone exceeding the prosthesis was recorded as positive, and the opposite was recorded as negative. The specific measurements are listed in Supplementary Tables 2, 3.

### Statistical Analysis

Grayscale data were read using Weasis version 3.7.1, and the length data of the overhang on each radial were read using Mimics. All data were retained to two decimal places. The means, standard deviations (SD), medians, and variability were calculated using SPSS version 26 (IBM Corp., Armonk, NY).

To minimize observational bias, two independent investigators repeated all imaging measurements at 2-week intervals. Intraclass correlation coefficients were used to assess intra- and interobserver reliabilities for all measurements. In this study, the intraclass correlation coefficient values for all measurements indicated intra- and interobserver reliabilities >0.8. Therefore, the mean values can be used for analysis.

## Results

### Radiographs

Postoperative radiographs showed that all bone fragments were cleared, and the joint space was rebalanced. Both the PEEK and HXLPE components showed low radiograph signals in the entire prosthetic knee system. Because of the low contrast, it was relatively difficult to distinguish the prosthesis from the soft tissue on the X-rays. However, the bonding of the cement between the articular prosthesis and the implant bed surface can be demonstrated because of the radiopaque nature of the bone cement. The knee prosthesis has a metal wire for positioning, which was clearly visible on radiographs and facilitated its intraoperative fluoroscopic examination (Figures 2A,B). Misalignment of the metal wires often indicates dislocation of the prosthesis (24).

### CT

CT with a window level of 350 HU and a window width of 2,000 HU allowed for clearer visualization of the prosthetic component. Most structures around the knee, including the PEEK and polyethylene components, as well as the femoral and tibial cement sleeves, could be reasonably well-visualized. We recommend that subsequent investigators look at the PEEK prosthetic knee system after implantation in this window width (Figures 2C,D).

The PEEK prosthesis value was 182.95 [SD 4.90, coefficient of variation (CV) 2.68] HU, the mean HXLPE value was −89.41 (SD 4.14, CV −4.63) HU, the lateral femoral osteochondral mean was 192.19 (SD 55.05, CV 28.64) HU, the lateral tibial osteochondral mean was 122.94 (SD 62.14, CV 42.86) HU, the medial femoral osteophyte mean was 180.76 (SD 43.48, CV 24.05) HU, and the medial tibial osteophyte mean was 282.59 (SD 69.28, CV 24.52) HU. After our initial exploration, we found that the range of variation in HU values for both HXLPE and PEEK materials in CT testing was in a relatively small range, as described in Supplementary Figure 10.

**Figure 6:**
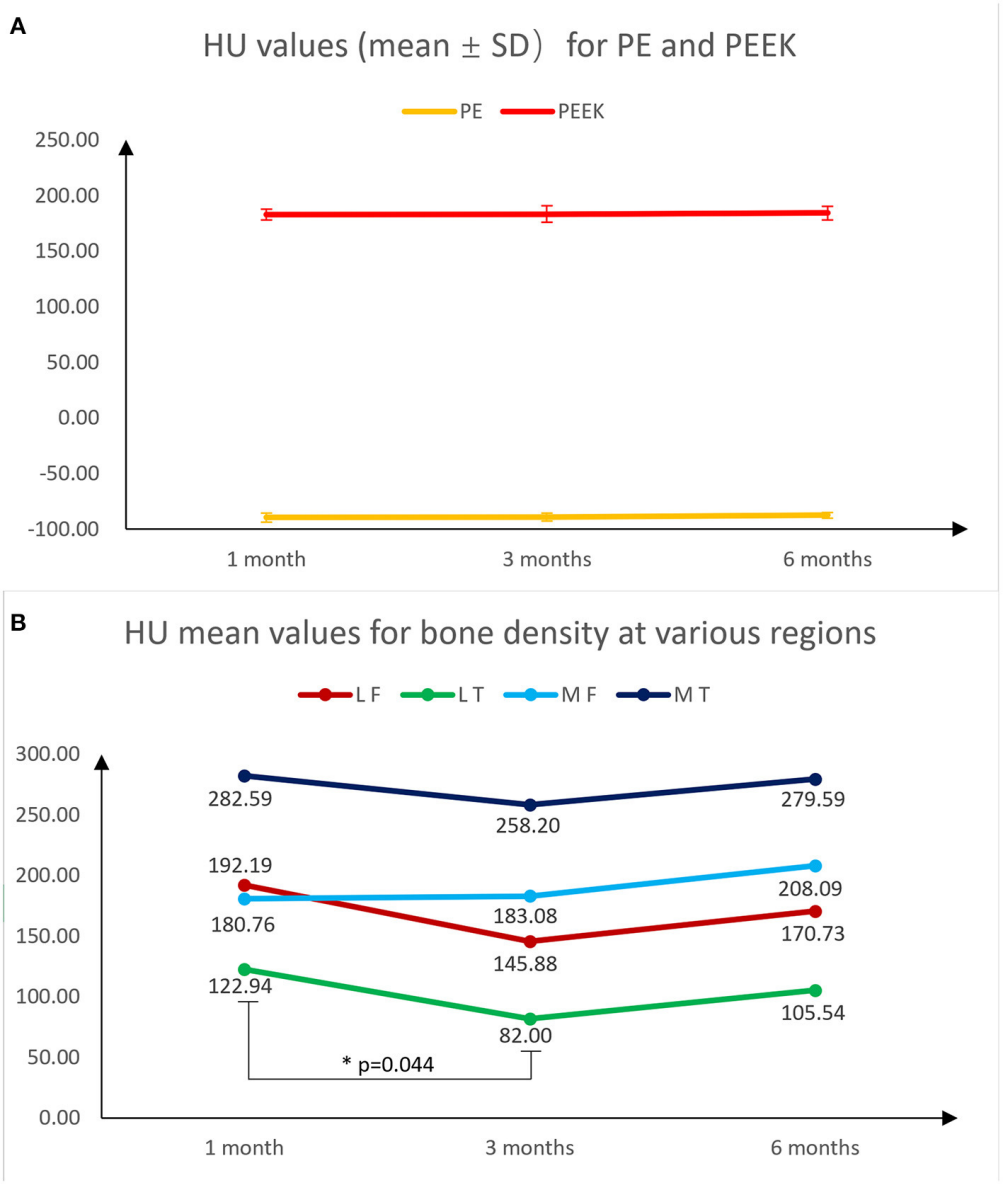
Fold plots of the change in HU values of PE, PEEK, lateral femur, lateral tibia, medial femur and medial tibia measured using CT at 1, 3, and 6 months postoperatively, which reflect the trend of density change in the measured area. Among them, the mean and standard deviation of HU values for PE and PEEK were almost unchanged **(A)**. Except for the medial femur, which did not decrease at 3 months, there was a trend of decreasing bone density at 3 months in the remaining regions, with a statistically significant decrease in the medial tibia. All regions showed a rebound in bone density at 6 months **(B)**.

Without the interference of metal artifacts and bone cement, the HU values of PEEK and HXLPE on CT had low inter-patient variability and were comparable, whereas the osteophyte density was more labile. The mean, SD, and variability of 1 month post operation data for all patients are presented in Table 1. The 3 and 6 months post operation bone density data are presented in Supplementary Tables 2, 3. In Supplementary Table 4, the results of the ANOVA analysis show that there was no significant difference in the overall CT HU value measurements of PE, PEEK, and bone density data we measured from 1 to 6 months, but there was a statistically significant difference in the decrease in bone density of the lateral tibial plateau by 3 months (*p* = 0.044), and there is no longer a significant difference in bone density between 6 and 3 months postoperatively (Figure 6, Supplementary Figures 11A–F). We are only able to provide partial 6-month follow-up information because the time for follow-up is not yet available. We also found that bone density decreased at 3 months and slightly rebounded at 6 months in all regions, except for the medial femoral BMD, which increased at 6 months, but none of them were statistically different. These results indicated that the CT HU values of the prosthesis components could be used as a reference in subsequent examinations to assess the patient's postoperative acute bone loss.

**Table 1:** Results of HU measurements for HXLPE, PEEK, and cancellous bone (1 month post operation).

**No**.	**PE**	**T PEEK**	**L F**	**L T**	**M F**	**M T**
1	−95.50	177.90	176.90	174.50	174.60	421.40
2	−91.50	185.20	214.90	117.50	139.50	252.10
3	−92.50	180.40	167.60	110.40	112.50	168.50
4	−92.30	176.10	169.70	132.70	166.40	227.60
5	−92.30	177.20	252.30	176.80	192.60	301.60
6	−86.10	190.60	256.10	139.00	270.90	310.80
7	−81.80	187.00	276.00	147.20	222.40	342.70
8	−87.70	183.20	164.10	72.10	164.10	292.60
9	−83.90	184.20	129.00	93.40	190.30	270.00
10	−90.50	187.70	115.30	65.80	174.30	238.60
Average	−89.41	182.95	192.19	122.94	180.76	282.59
SD	4.14	4.90	55.05	38.60	43.48	69.28
C.V.	−4.63	2.68	28.64	31.40	24.05	24.52

*HU values for HXLPE, PEEK, lateral femur, lateral tibia, medial femur, and medial tibial cancellous bone were measured on CT. The measurement method is shown in Figure 3. HU, Hounsfield unit; HXLPE, highly cross-linked polyethylene; PEEK, polyetheretherketone; F, femoral; T, tibial; SD, standard deviation; CV, coefficient of variation; CT, computed tomography*.

### 3D Model Reconstruction

The 3D reconstruction model provides a broad view of the frontal, posterior, lateral, and medial aspects of the model. The 3D model provides a cursory view of the prosthetic alignment and generally shows the position of the implant and the match between the prosthetic components. The model reconstruction showed that the components of the prosthetic knee system were well-mounted and accurately aligned with the HXLPE bearing in place and the patella and patellar prosthesis in place (Figures 2E–H).

### Overhang Measurement

The specific measurement method for the overhang of the prostheses has been described in detail in Section Radiographic Evaluation. Before the measurement, the prosthesis within the reconstruction model was replaced by an engineering prosthesis model to obtain more accurate edge measurements.

The overhang was measured on the femoral side by taking the length of the prosthesis and bone-implant bed on the horizontal line of the five plane turning points of the prosthesis, the midpoint of each plane, and the position of the inner and outer lateral edges of the upper edge of the prosthesis (25). Greater than 3 mm on the femoral side and 1.5 mm on the tibial side were defined as overhang, and vice versa as underhang (25, 26). The specific measurement for 1, 3, and 6 months post operation results are shown in Supplementary Tables 5–10. The results of the ANOVA analysis showed that there were no explicit that differences in the measurements at 1, 3, and 6 months, and the results are shown in Supplementary Table 11 (*p* > 0.05). For reasons previously stated, only partial measurements at 6 months are available.

We did not observe any overhang on the femur bone-implant interface. Two cases of overhang were observed on the tibia bone-implant interface in which case 3 had an overhang in both the medial posterior and lateral posterior angles (2.72 and 2.26 mm, respectively), whereas case 7 had a 4.04-mm overhang in the medial posterior angle. Figure 4 shows a 3D model of a patient with significant overhang at the medial posterior tibial plateau, which was obtained from the postoperative CT image reconstruction of case 7.

## Discussion

Admittedly, as a very early report of a new technology, this study has some limitations. We included only a small number of participants and no subgroup analyses were performed, such as differences in sex, age and BMI. The density of cement and bone on CT images were similar, making it difficult to distinguish between bone volume and overhang after model reconstruction. During densitometry, the density of the PEEK prosthesis close to the cemented area was prone to shift. As the first clinical trial of a fully assembled PEEK artificial knee prosthesis, the short follow-up period prevented us from evaluating imaging and other aspects of the patient's mid- to late-stage condition. However, our results demonstrate the importance of CT for the postoperative assessment of PEEK prosthetic knees.

As a copolymer compound, in contrast to metals, the biomaterial PEEK has a range of properties superior to those of metals, including reduced allergenicity, lighter weight (27), greater fatigue and chemical resistance (28). The average amount of bone removed in a total knee arthroplasty is about 155 g, while the weight of a metal knee prosthesis with bone cement is about 430 g, so the weight gain of the knee joint after implantation of a metal joint is very significant (29). According to the data provided by the manufacturer, the weight of our prosthesis with bone cement is ~120 g. Attempts to use polymers for joint prostheses began in the 1980's, with Moore et al. first pioneering attempts to use polyacetal (Delrin) for femoral prostheses (30). However, due to the poor sterilization tolerance of Delrin and its poor fixation stability to bone, research on polyacetal prostheses has since declined. The leaching of formaldehyde is also a hindrance to its promotion for long-term application *in vivo* (31). The structure of PEEK as a polyetheretherketone gives it excellent chemical resistance, making it extremely inactive and inherently resistant to chemical, thermal, and post-irradiation degradation. Radiation stability data shows that PEEK components can be effectively sterilized by gamma irradiation in air (28). Smooth PEEK prostheses are weakly osseointegrated and require surface modification for osteoinduction to enhance osseointegration, making biofixation of pure PEEK prostheses more difficult to achieve (32). Our choice of a cemented prosthesis in this experiment circumvents this problem and maintains consistency with conventional knee prosthesis fixation methods. Carbon fiber-reinforced PEEK has also been a hot topic of research in the past, but research in this area is slowly declining due to the higher polyethylene wear and still-higher modulus of elasticity associated with carbon fibers (33). We conducted the first-in-human trial of the world's first totally modular PEEK TKA and performed X-ray, CT, and measurement studies on early prosthesis position, peripheral bone density, cement fixation status, and prosthesis-bone border matching after PEEK implantation.

Subjective patient satisfaction after TKA is often lower than orthopedic surgeons' satisfaction with the imaging assessment, and prosthetic overhang and soft-tissue impingement have been suggested as potential causes of postoperative pain and decreased ROM (25, 34, 35). Previous studies have reported that the overhang in knee replacement prostheses may lead to surrounding soft-tissue compression, resulting in pain and poor functional recovery in the medium to long term postoperatively (25, 36–38). Simultaneously, underhang may be associated with tibial osteolysis and prosthesis sinking (39). Current techniques are limited in measuring the impingement site because metal prostheses produce artifacts in CT examinations. Moreover, most overhang measurements after prosthesis fitting are mostly performed in a two-dimensional plane (39), which undoubtedly produces errors in 3D examinations. The projections of both metal and bone are compressed in a finite number of two planes, and that the change in projection due to rotation causes many times many times loosening may be underestimated (39). Our study is the first to use unprocessed raw data for femoral and tibial overhang measurements due to the use of a PEEK prosthetic system without metal CT artifacts.

Mahoney et al. suggested that a >3-mm overhang at any one or more locations of the lateral femoral prosthesis increased the probability of postoperative pain by 90% after 2 years postoperatively (25). Moreover, we set overhangs of >3 mm as a risk factor in our study. However, no >3-mm overhang was observed in this study. No significant overhang was found on the femoral side (≤ 1.69 mm). On average, the femoral and tibial prostheses were slightly smaller than the osteotomy plane in the measurement direction, with an average overhang of 1.28 and 1.32 mm on the femoral and tibial sides, respectively. The tibial overhang generally occurs at the posterior corner of the tibial osteotomy surface, and the average overhang is 2.4 mm according to the ideal position (26). However, in this study, two patients had overhangs (2.72 and 4.04 mm), and the prosthesis was clearly outside of the osteotomy plane in the 3D model. Moreover, we observed that the underhang was relatively common on the femoral (up to 9.14 mm) and tibial (up to 7.15 mm) sides at the posterior lateral tibial angle. The femoral side had at least one measurement point of underhang in seven cases and more than three measurement points of underhang in four cases, with the largest degree of underhang occurring at point 4 in case 7 at 9.14 mm. The presence of underhang in conventional prostheses correlates with the degree of bone resorption in the corresponding tibia (39, 40), and the use of PEEK prostheses may reduce the occurrence of this phenomenon. These results may guide prosthetic fitting techniques and prosthesis design to reduce soft-tissue impingement and improve postoperative satisfaction in patients who had undergone TKA.

As an another issue of concern, although periprosthetic bone and stress-masking effects do not affect short-term patient satisfaction after TKA, they are a concern in the long term. Long-term osteoporosis not only affects prosthesis longevity but may also increase the risk of periprosthetic fractures (41). Biological fixation has been proposed as an alternative to cemented fixation to reduce osteoporosis following long-term periprosthetic fixation. Conversely, bone density around metal prostheses is difficult to measure, making it impossible to quantitatively analyze the relationship between periprosthetic bone density and late prosthetic failure. Although dual-energy radiography can measure whole-body bone density, it does not represent actual bone density changes in the prosthesis area. A more established method of measuring bone density in specific areas is based on quantitative CT (42, 43). In the past, this method could not be used in patients after TKA because of metal artifacts, but the PEEK prosthesis solves this problem. In this study, the HU values of PEEK, HXLPE, and cancellous bone in the stress concentration area were measured on CT scans. The density values of PEEK and HXLPE were very stable on CT (mean: −89.41 and 182.95 HU, CV −4.63 and 2.68%, respectively) and could be used as a reference value to quantify the bone loss of patients during mid- and long-term postoperative examinations (44), a quantitative localization that is not possible with conventional metal prostheses.

To avoid errors between CT examinations, we sought data that could be used as a reference value on CT images. We found that both PEEK prosthesis and HXLPE bearing maintained low variability between CT examinations. Moreover, we may consider setting parameters to normalize the bone mineral density (BMD) using the grayscale values of PEEK or HXLPE in the future, thus avoiding errors in the examination that may mislead the trend in BMD. Such a method would be useful for the prevention, diagnosis, treatment, and intervention of postoperative osteoporosis in artificial joints, preventing prosthetic loosening and periprosthetic fractures.

In conclusion, the first clinical application of the totally modular PEEK prosthetic joint is a milestone in developing a full-polymer total knee replacement system, offering a better option for patients with metal allergies. The method of local precision assessment may theoretically improve the level and accuracy of imaging assessment of patients after TKA. However, the long-term clinical performance and patient satisfaction of all-polymer joints after implantation in humans still require longer follow-up.

## Data Availability Statement

The raw data supporting the conclusions of this article will be made available by the authors, without undue reservation.

## Ethics Statement

The studies involving human participants were reviewed and approved by the Shanghai Jiao Tong University School of Medicine, Renji Hospital Ethics Committee (KY2021-025). Written informed consent was obtained from individual or guardian participants. The patients/participants provided their written informed consent to participate in this study.

## Author Contributions

YW primarily contributed to the study conception and design. ZC, XQ, and YZ performed material preparation, data collection, and analysis. ZC wrote the first draft of the manuscript. All authors commented on previous versions of the manuscript and read and approved the final manuscript.

## Funding

This work was supported by the National Key R&D Program of China (Grant No. 016YFC1101802).

## Conflict of Interest

The authors declare that the research was conducted in the absence of any commercial or financial relationships that could be construed as a potential conflict of interest.

## Publisher's Note

All claims expressed in this article are solely those of the authors and do not necessarily represent those of their affiliated organizations, or those of the publisher, the editors and the reviewers. Any product that may be evaluated in this article, or claim that may be made by its manufacturer, is not guaranteed or endorsed by the publisher.

## Abbreviations

3D: three-dimensional
BMD: bone mineral density
BMI: body mass index
CT: computed tomography
CV: coefficient of variation
HU: Hounsfield unit
HXLPE: highly cross-linked polyethylene
MRI: magnetic resonance imaging
PEEK: polyetheretherketone
ROI: region-of-interest
ROM: range of motion
SD: standard deviation
TKA: total knee arthroplasty.
