# Supplementary material for: Preliminary Study on Immediate Postoperative CT Images and Values of the Modular Polyetheretherketone Based Total Knee Arthroplasty: An Observational First-in-Human Trial
Source: Front Surg. 2022 Feb 14;9:809699. doi: 10.3389/fsurg.2022.809699 (PMC8882580; doi:10.3389/fsurg.2022.809699)
Supplement: Supplementary file 1 [file Data_Sheet_1.docx]

Supplement materials

Preliminary study on immediate postoperative CT images and values of the modular polyetheretherketone based total knee arthroplasty: an observational first-in-human trial

**Table. S1 Patient demographic data**

|  | **Age** | **sex** | **height(cm)** | **weight(kg)** | **BMI(kg/m**^2^**)** | **limb** |
| --- | --- | --- | --- | --- | --- | --- |
| 1 | 64 | M | 175 | 53 | 17.31 | Right |
| 2 | 68 | F | 156 | 61 | 25.07 | Left |
| 3 | 66 | F | 159 | 57 | 22.55 | Right |
| 4 | 62 | F | 163 | 58 | 21.83 | Right |
| 5 | 70 | F | 148 | 67 | 30.59 | Right |
| 6 | 64 | F | 158 | 60 | 24.03 | Right |
| 7 | 59 | F | 160 | 64 | 25.00 | Left |
| 8 | 69 | F | 163 | 89 | 33.50 | Left |
| 9 | 74 | F | 150 | 55 | 24.44 | Right |
| 10 | 73 | F | 152 | 60 | 25.97 | Left |
| Average | 66.9 |  | 158.4 | 62.4 | 24.87 |  |
| Median | 67 |  | 158.5 | 60 | 24.72 |  |

# All relevant data were collected on the day of admission and are preoperative. BMI, body mass index; M, male; F, female

**Table. S2 Results of HU measurements for HXLPE, PEEK, and cancellous bone (3 months post operation)**

| NO. | PE | T PEEK | L F | L T | M F | M T |
| --- | --- | --- | --- | --- | --- | --- |
| 1 | -91.40 | 178.40 | 176.40 | 159.10 | 197.60 | 427.40 |
| 2 | -89.30 | 180.50 | 194.40 | 145.90 | 194.40 | 240.70 |
| 3 | -90.20 | 179.20 | 66.20 | 45.00 | 245.10 | 175.30 |
| 4 | -95.20 | 180.60 | 157.60 | 103.10 | 133.00 | 205.20 |
| 5 | -82.30 | 178.70 | 209.20 | 77.00 | 203.50 | 212.20 |
| 6 | -89.20 | 198.50 | 186.60 | 104.00 | 202.30 | 166.50 |
| 7 | -85.10 | 184.80 | 224.80 | 98.20 | 181.80 | 410.20 |
| 8 | -84.40 | 174.30 | 135.90 | 47.30 | 151.90 | 257.40 |
| 9 | -91.70 | 193.20 | 63.90 | 49.90 | 206.00 | 261.70 |
| 10 | -91.70 | 184.90 | 43.80 | -9.50 | 115.20 | 225.40 |
| average | -89.05 | 183.31 | 145.88 | 82.00 | 183.08 | 258.20 |
| sd | 3.76 | 7.39 | 65.75 | 50.79 | 38.85 | 90.27 |
| C.V. | -4.23 | 4.03 | 45.07 | 61.94 | 21.22 | 34.96 |

**Table. S3 Results of HU measurements for HXLPE, PEEK, and cancellous bone (6 months post operation)**

| NO. | PE | T PEEK | L F | L T | M F | M T |
| --- | --- | --- | --- | --- | --- | --- |
| 1 | -89.70 | 184.50 | 154.40 | 156.40 | 225.50 | 429.00 |
| 2 | -88.90 | 180.60 | 206.80 | 117.00 | 156.30 | 290.90 |
| 3 | -87.40 | 173.90 | 73.20 | 51.30 | 321.80 | 195.70 |
| 4 | -91.80 | 188.50 | 154.90 | 102.80 | 138.10 | 192.40 |
| 5 | -86.00 | 181.70 | 192.20 | 68.90 | 195.80 | 186.90 |
| 6 | -85.90 | 190.40 | 174.00 | 108.90 | 186.50 | 238.40 |
| 7 | -82.90 | 189.90 | 239.60 | 133.50 | 232.60 | 423.80 |
| average | -87.51 | 184.21 | 170.73 | 105.54 | 208.09 | 279.59 |
| sd | 2.71 | 5.98 | 52.51 | 36.06 | 60.60 | 106.60 |
| C.V. | -3.09 | 3.25 | 30.76 | 34.17 | 29.12 | 38.13 |

**Table. S4 ANOVA analysis test for HU measurements for HXLPE, PEEK, and cancellous bone (1, 3 and 6 months post operation)**

|  | | Sum of Squares | Mean Square | Sig. |
| --- | --- | --- | --- | --- |
| PE | Between Groups | 15.912 | 7.956 | 0.598 |
|  | Within Groups | 363.903 | 15.163 |  |
|  | Total | 379.814 |  |  |
| PEEK | Between Groups | 6.744 | 3.372 | 0.916 |
|  | Within Groups | 922.943 | 38.456 |  |
|  | Total | 929.687 |  |  |
| LF | Between Groups | 10737.953 | 5368.976 | 0.231 |
|  | Within Groups | 82729.519 | 3447.063 |  |
|  | Total | 93467.472 |  |  |
| LT | Between Groups | 8429.379 | 4214.689 | 0.124 |
|  | Within Groups | 44433.321 | 1851.388 |  |
|  | Total | 52862.700 |  |  |
| MF | Between Groups | 3576.921 | 1788.461 | 0.454 |
|  | Within Groups | 52634.049 | 2193.085 |  |
|  | Total | 56210.970 |  |  |
| MT | Between Groups | 3412.349 | 1706.175 | 0.803 |
|  | Within Groups | 184711.898 | 7696.329 |  |
|  | Total | 188124.247 |  |  |

| **Multiple Comparisons** | | | | | |
| --- | --- | --- | --- | --- | --- |
| Dependent Variable | Month | | Mean Difference (I-J) | Std. Error | Sig. |
|  |  |  |  |  |  |
| PE | 1 | 3 | -0.36000 | 1.74141 | 0.838 |
|  |  | 6 | -1.89571 | 1.91894 | 0.333 |
|  | 3 | 1 | 0.36000 | 1.74141 | 0.838 |
|  |  | 6 | -1.53571 | 1.91894 | 0.431 |
|  | 6 | 1 | 1.89571 | 1.91894 | 0.333 |
|  |  | 3 | 1.53571 | 1.91894 | 0.431 |
| PEEK | 1 | 3 | -0.36000 | 2.77330 | 0.898 |
|  |  | 6 | -1.26429 | 3.05603 | 0.683 |
|  | 3 | 1 | 0.36000 | 2.77330 | 0.898 |
|  |  | 6 | -0.90429 | 3.05603 | 0.770 |
|  | 6 | 1 | 1.26429 | 3.05603 | 0.683 |
|  |  | 3 | 0.90429 | 3.05603 | 0.770 |
| LF | 1 | 3 | 46.31000 | 26.25667 | 0.091 |
|  |  | 6 | 21.46143 | 28.93344 | 0.465 |
|  | 3 | 1 | -46.31000 | 26.25667 | 0.091 |
|  |  | 6 | -24.84857 | 28.93344 | 0.399 |
|  | 6 | 1 | -21.46143 | 28.93344 | 0.465 |
|  |  | 3 | 24.84857 | 28.93344 | 0.399 |
| LT | 1 | 3 | 40.94000^*^ | 19.24260 | 0.044 |
|  |  | 6 | 17.39714 | 21.20431 | 0.420 |
|  | 3 | 1 | -40.94000^*^ | 19.24260 | 0.044 |
|  |  | 6 | -23.54286 | 21.20431 | 0.278 |
|  | 6 | 1 | -17.39714 | 21.20431 | 0.420 |
|  |  | 3 | 23.54286 | 21.20431 | 0.278 |
| MF | 1 | 3 | -2.32000 | 20.94319 | 0.913 |
|  |  | 6 | -27.32571 | 23.07827 | 0.248 |
|  | 3 | 1 | 2.32000 | 20.94319 | 0.913 |
|  |  | 6 | -25.00571 | 23.07827 | 0.289 |
|  | 6 | 1 | 27.32571 | 23.07827 | 0.248 |
|  |  | 3 | 25.00571 | 23.07827 | 0.289 |
| MT | 1 | 3 | 24.39000 | 39.23348 | 0.540 |
|  |  | 6 | 3.00429 | 43.23319 | 0.945 |
|  | 3 | 1 | -24.39000 | 39.23348 | 0.540 |
|  |  | 6 | -21.38571 | 43.23319 | 0.625 |
|  | 6 | 1 | -3.00429 | 43.23319 | 0.945 |
|  |  | 3 | 21.38571 | 43.23319 | 0.625 |
| *. The mean difference is significant at the 0.05 level. | | | | | |

**Table. S5 Measurements of the femoral overhang. The method of taking points from point 1 to point 13 is described in Figure 4a,b. The units in the table are in millimeters (1 month post operation).**

|  | 1 | 2 | 3 | 4 | 5 | 6 | 7 | 8 | 9 | 10 | 11 | 12 | 13 |
| --- | --- | --- | --- | --- | --- | --- | --- | --- | --- | --- | --- | --- | --- |
| 1 | 7.98 | 7.97 | 8.43 | 8.86 | 6.84 | 6.02 | 1.85 | 0.78 | 0.36 | 0.08 | -0.35 | 0.89 | 0.38 |
| 2 | 0.22 | 2.83 | 3.79 | 3.73 | 3.10 | 2.22 | 0.30 | -0.17 | 1.06 | 1.26 | 0.61 | 2.62 | 0.70 |
| 3 | -0.19 | 0.20 | 2.24 | -0.08 | -0.37 | 0.63 | 1.90 | 0.11 | 2.19 | 1.75 | 1.28 | 2.41 | 2.05 |
| 4 | 0.53 | 0.67 | 2.52 | 0.77 | 0.91 | 1.71 | 2.60 | 0.07 | 0.18 | -0.31 | -0.03 | 0.29 | 1.01 |
| 5 | 0.41 | 0.50 | 2.04 | 0.98 | 1.88 | 1.17 | 3.72 | 1.49 | 2.58 | 3.96 | 3.80 | 3.18 | 1.42 |
| 6 | 3.76 | 1.22 | 1.21 | 1.08 | 0.52 | 0.58 | 1.29 | 0.56 | 1.69 | 1.65 | 0.62 | 1.37 | -0.61 |
| 7 | 2.28 | 8.93 | 3.74 | 9.14 | 6.23 | 0.33 | 0.85 | 1.94 | 1.48 | 2.29 | 2.52 | 1.31 | -1.69 |
| 8 | 0.10 | 2.22 | 3.68 | 3.47 | 2.68 | 2.46 | 1.03 | 0.21 | 1.27 | 2.10 | 1.15 | 1.58 | 0.25 |
| 9 | 0.42 | 2.95 | 3.89 | 3.43 | 1.20 | 0.36 | 0.51 | 0.47 | 1.57 | 1.69 | 1.80 | 2.02 | -0.66 |
| 10 | 0.02 | 0.81 | 3.10 | 1.78 | 1.00 | 1.45 | -0.32 | 0.00 | -1.30 | 0.39 | 2.26 | 1.14 | 2.19 |
| Mean | 1.87 | 2.27 | 1.24 | 2.41 | 1.85 | 1.13 | 0.92 | 0.52 | 0.83 | 0.90 | 0.98 | 0.70 | 0.97 |
| SD | 2.57 | 3.12 | 1.96 | 3.26 | 2.40 | 1.70 | 1.19 | 0.69 | 1.12 | 1.24 | 1.26 | 0.87 | 1.24 |

**Table. S6 Measurements of the tibial overhang. The method of taking points from point 14 to point 13 is described in Figure 4c (1 month post operation).**

|  | 14 | 15 | 16 | 17 | 18 | 19 | 20 | 21 | 22 | 23 |
| --- | --- | --- | --- | --- | --- | --- | --- | --- | --- | --- |
| 1 | 4.53 | 4.65 | 2.52 | 2.35 | 1.55 | 0.12 | 2.07 | 0.36 | 1.83 | 6.23 |
| 2 | 1.11 | 4.18 | 2.84 | 0.28 | -0.03 | -0.01 | 2.15 | 1.24 | 2.66 | 0.61 |
| 3 | -2.72 | 0.25 | 1.20 | 0.36 | 1.62 | 1.90 | 1.80 | 1.99 | -0.65 | -2.26 |
| 4 | 1.25 | 0.85 | 0.43 | 2.53 | 2.86 | 2.03 | 3.50 | 3.42 | 1.74 | 0.98 |
| 5 | -0.37 | 0.00 | 0.29 | 2.34 | 0.26 | -0.06 | 3.91 | 2.06 | 0.54 | 0.64 |
| 6 | 0.93 | 0.96 | 2.03 | 1.47 | 1.53 | 0.41 | 1.93 | 3.02 | 2.55 | 2.84 |
| 7 | -1.03 | -4.04 | -0.86 | 2.16 | 2.72 | 3.41 | 3.05 | 6.50 | 7.27 | 3.15 |
| 8 | 0.28 | 0.36 | 0.99 | 0.58 | 0.19 | 0.64 | 4.54 | 7.15 | 6.96 | 1.14 |
| 9 | 0.75 | -0.02 | 0.60 | 2.46 | 0.93 | 2.35 | 1.00 | 1.39 | 1.28 | -0.55 |
| 10 | 0.01 | -0.41 | -0.34 | -0.70 | 1.33 | 2.86 | 1.73 | 5.38 | 2.48 | 3.78 |
| mean | 1.24 | 1.59 | 0.95 | 1.00 | 0.77 | 1.15 | 0.95 | 1.89 | 1.78 | 1.88 |
| sd | 1.86 | 2.42 | 1.20 | 1.16 | 0.99 | 1.29 | 1.12 | 2.34 | 2.55 | 2.41 |

**Table. S7 Measurements of the femoral overhang. The method of taking points from point 1 to point 13 is described in Figure 4a,b. The units in the table are in millimeters (3 months post operation).**

|  | 1 | 2 | 3 | 4 | 5 | 6 | 7 | 8 | 9 | 10 | 11 | 12 | 13 |
| --- | --- | --- | --- | --- | --- | --- | --- | --- | --- | --- | --- | --- | --- |
| 1 | 6.73 | 9.93 | 8.66 | 8.18 | 6.31 | 3.46 | 1.52 | -2.40 | -1.35 | 1.77 | -1.04 | -2.03 | -0.46 |
| 2 | 0.82 | 1.02 | 6.56 | 3.20 | 3.39 | 5.78 | 0.06 | 2.60 | -0.82 | 3.07 | 1.86 | 1.28 | 1.29 |
| 3 | 1.17 | 1.08 | 5.22 | 1.67 | 0.65 | -2.18 | 2.14 | 1.07 | 2.12 | 3.64 | 1.71 | 7.09 | 1.14 |
| 4 | -1.83 | 3.33 | 0.50 | -1.40 | 2.55 | 2.03 | 2.87 | 1.52 | -0.36 | 5.45 | 1.72 | 1.37 | 0.21 |
| 5 | 1.17 | 1.08 | 5.22 | 1.67 | 0.65 | -2.18 | 2.14 | 1.07 | 2.12 | 3.64 | 1.71 | 7.09 | 1.14 |
| 6 | 6.32 | -0.23 | -0.08 | 3.31 | 3.47 | -1.86 | 1.35 | -3.44 | -2.38 | 0.72 | -0.01 | 6.38 | -2.18 |
| 7 | 2.12 | 11.09 | 4.53 | 10.84 | 6.23 | -0.25 | -1.50 | 2.14 | -0.36 | 3.26 | 1.96 | 4.51 | -1.75 |
| 8 | 1.70 | 1.55 | 5.38 | 1.91 | 2.27 | 0.19 | 0.70 | 3.20 | 3.24 | -0.31 | 1.71 | -3.99 | -3.88 |
| 9 | -0.55 | 2.36 | 3.20 | 5.74 | 2.57 | -0.06 | 0.94 | -2.48 | -3.06 | 0.58 | 1.12 | 7.94 | 1.49 |
| 10 | 1.93 | 0.11 | 4.41 | 2.42 | 2.09 | 1.77 | -1.80 | 1.72 | 0.25 | -1.19 | 3.74 | -0.41 | 1.79 |
| Mean | 1.96 | 3.13 | 4.36 | 3.75 | 3.02 | 0.67 | 0.84 | 0.50 | -0.06 | 2.06 | 1.45 | 2.92 | -0.12 |
| SD | 2.69 | 4.03 | 2.62 | 3.57 | 1.96 | 2.61 | 1.54 | 2.37 | 2.03 | 2.09 | 1.26 | 4.26 | 1.91 |

**Table. S8 Measurements of the tibial overhang. The method of taking points from point 14 to point 13 is described in Figure 4c at (3 months post operation).**

|  | 14 | 15 | 16 | 17 | 18 | 19 | 20 | 21 | 22 | 23 |
| --- | --- | --- | --- | --- | --- | --- | --- | --- | --- | --- |
| 1 | 4.37 | 4.30 | 2.41 | 2.32 | 3.00 | 0.59 | 3.41 | 0.43 | 1.69 | 7.30 |
| 2 | 1.40 | 4.36 | 4.46 | -0.90 | -1.92 | 1.69 | 3.83 | 1.50 | 2.18 | -0.66 |
| 3 | 0.04 | 0.23 | 0.13 | 2.13 | 1.66 | 0.06 | 5.50 | 1.79 | 2.30 | 1.53 |
| 4 | 2.13 | 0.09 | -0.62 | 2.05 | 4.68 | 1.98 | 3.91 | 3.63 | 2.93 | 2.31 |
| 5 | 0.04 | 0.23 | 0.13 | 2.13 | 1.66 | 0.06 | 5.50 | 1.79 | 2.30 | 1.53 |
| 6 | 0.43 | 1.84 | 1.92 | 2.43 | 2.19 | -0.35 | 2.10 | 4.07 | 1.84 | 2.48 |
| 7 | -1.89 | -4.31 | -1.91 | 2.18 | 2.92 | 2.96 | 1.43 | 5.81 | 6.30 | 3.92 |
| 8 | 1.92 | 0.86 | 0.96 | -0.25 | -0.17 | -1.06 | 2.38 | 6.81 | 6.06 | 1.37 |
| 9 | -0.05 | -0.19 | 0.69 | 0.76 | 1.00 | 1.48 | 0.66 | 1.22 | 0.41 | 0.84 |
| 10 | -0.57 | -0.44 | -0.90 | -1.03 | 2.17 | 1.72 | 1.38 | 5.17 | 1.74 | 3.87 |
| mean | 0.78 | 0.70 | 0.73 | 1.18 | 1.72 | 0.91 | 3.01 | 3.22 | 2.78 | 2.45 |
| sd | 1.74 | 2.49 | 1.84 | 1.41 | 1.81 | 1.24 | 1.70 | 2.19 | 1.91 | 2.18 |

**Table. S9 Measurements of the femoral overhang. The method of taking points from point 1 to point 23 is described in Figure 4a,b. The units in the table are in millimeters (6 months post operation).**

|  | 1 | 2 | 3 | 4 | 5 | 6 | 7 | 8 | 9 | 10 | 11 | 12 | 13 |
| --- | --- | --- | --- | --- | --- | --- | --- | --- | --- | --- | --- | --- | --- |
| 1 | 7.99 | 8.78 | 6.28 | 7.96 | 4.06 | 5.52 | 0.29 | 4.55 | 0.22 | 0.10 | 0.11 | -1.96 | -5.01 |
| 2 | -1.33 | 4.06 | 1.19 | 3.18 | 1.56 | -1.13 | -2.84 | 4.16 | -0.49 | 3.18 | -0.33 | 2.45 | -3.21 |
| 3 | 0.09 | 3.49 | 2.60 | 0.75 | -1.33 | -2.22 | 4.56 | 1.73 | 3.54 | -2.44 | 3.52 | 4.21 | 8.36 |
| 4 | 1.49 | 1.19 | 2.61 | 0.37 | 1.85 | -1.16 | 6.99 | 0.05 | 4.55 | 2.48 | -0.87 | 4.80 | -0.96 |
| 5 | -0.94 | 4.25 | 4.67 | 3.31 | 2.73 | 1.92 | 0.97 | -0.77 | 1.39 | 3.78 | 0.29 | 2.31 | 1.04 |
| 6 | -0.94 | 4.25 | 4.67 | 3.31 | 2.73 | 1.92 | 0.97 | -0.77 | 1.39 | 3.78 | 0.29 | 2.31 | 1.04 |
| 7 | -1.85 | 0.54 | 2.81 | 0.55 | 1.86 | 0.58 | 0.23 | 3.39 | 1.55 | 5.90 | 3.67 | 2.41 | 1.91 |
| mean | 0.75 | 5.02 | 3.96 | 4.00 | 2.39 | 1.10 | 1.05 | 2.02 | 1.14 | 1.92 | 1.27 | 2.28 | 0.57 |
| sd | 3.46 | 3.20 | 1.81 | 3.27 | 2.05 | 2.49 | 2.35 | 2.18 | 1.30 | 3.01 | 1.67 | 2.05 | 4.26 |

**Table. S10 Measurements of the tibial overhang. The method of taking points from point 14 to point 13 is described in Figure 4c at (6 months post operation).**

|  | 14 | 15 | 16 | 17 | 18 | 19 | 20 | 21 | 22 | 23 |
| --- | --- | --- | --- | --- | --- | --- | --- | --- | --- | --- |
| 1 | 4.49 | 5.38 | 4.04 | 2.42 | 3.28 | 1.18 | 3.96 | -1.17 | 0.46 | 5.82 |
| 2 | 0.95 | 6.54 | 3.59 | -2.03 | -2.45 | 0.24 | 2.08 | 1.89 | 3.71 | 0.77 |
| 3 | -3.40 | 0.61 | 1.69 | 0.39 | 2.16 | 1.68 | 0.57 | 3.85 | -1.33 | -2.20 |
| 4 | 2.18 | 2.46 | 1.59 | 0.65 | 2.03 | 2.17 | 4.00 | 3.21 | 1.17 | 0.79 |
| 5 | 0.56 | 0.86 | -0.08 | 0.37 | -0.77 | -0.92 | 4.75 | 8.34 | 8.80 | 1.24 |
| 6 | 0.56 | 0.86 | -0.08 | 0.37 | -0.77 | -0.92 | 4.75 | 8.34 | 8.80 | 1.24 |
| 7 | 2.27 | 0.15 | 0.77 | 2.49 | 0.75 | 1.90 | 0.74 | -0.03 | 0.62 | -1.40 |
| mean | 0.58 | 1.38 | 1.41 | 1.02 | 0.50 | 0.98 | 3.03 | 4.02 | 3.99 | 1.37 |
| sd | 2.51 | 3.71 | 1.77 | 1.78 | 1.96 | 1.65 | 1.86 | 3.96 | 4.22 | 2.84 |

**Table. S11 ANOVA analysis for the overhang measurements from point 1-23 (1，3 and 6 months post operation**)

| Month |  | Sum of Squares | Mean Square | Sig. |
| --- | --- | --- | --- | --- |
| 1 | Between Groups | 6.033 | 3.016 | 0.696 |
|  | Within Groups | 196.589 | 8.191 |  |
|  | Total | 202.622 |  |  |
| 2 | Between Groups | 22.014 | 11.007 | 0.422 |
|  | Within Groups | 294.917 | 12.288 |  |
|  | Total | 316.930 |  |  |
| 3 | Between Groups | 4.026 | 2.013 | 0.664 |
|  | Within Groups | 116.149 | 4.840 |  |
|  | Total | 120.175 |  |  |
| 4 | Between Groups | 2.080 | 1.040 | 0.913 |
|  | Within Groups | 274.547 | 11.439 |  |
|  | Total | 276.628 |  |  |
| 5 | Between Groups | 2.451 | 1.226 | 0.771 |
|  | Within Groups | 111.773 | 4.657 |  |
|  | Total | 114.224 |  |  |
| 6 | Between Groups | 5.270 | 2.635 | 0.608 |
|  | Within Groups | 124.284 | 5.178 |  |
|  | Total | 129.553 |  |  |
| 7 | Between Groups | 1.429 | 0.714 | 0.777 |
|  | Within Groups | 67.111 | 2.796 |  |
|  | Total | 68.540 |  |  |
| 8 | Between Groups | 11.653 | 5.826 | 0.207 |
|  | Within Groups | 83.153 | 3.465 |  |
|  | Total | 94.806 |  |  |
| 9 | Between Groups | 8.816 | 4.408 | 0.186 |
|  | Within Groups | 58.660 | 2.444 |  |
|  | Total | 67.476 |  |  |
| 10 | Between Groups | 1.770 | 0.885 | 0.822 |
|  | Within Groups | 107.441 | 4.477 |  |
|  | Total | 109.211 |  |  |
| 11 | Between Groups | 0.129 | 0.064 | 0.967 |
|  | Within Groups | 45.363 | 1.890 |  |
|  | Total | 45.492 |  |  |
| 12 | Between Groups | 7.717 | 3.859 | 0.628 |
|  | Within Groups | 195.142 | 8.131 |  |
|  | Total | 202.859 |  |  |
| 13 | Between Groups | 2.696 | 1.348 | 0.813 |
|  | Within Groups | 155.372 | 6.474 |  |
|  | Total | 158.068 |  |  |
| 14 | Between Groups | 0.484 | 0.242 | 0.942 |
|  | Within Groups | 96.194 | 4.008 |  |
|  | Total | 96.677 |  |  |
| 15 | Between Groups | 2.499 | 1.249 | 0.856 |
|  | Within Groups | 191.115 | 7.963 |  |
|  | Total | 193.614 |  |  |
| 16 | Between Groups | 1.938 | 0.969 | 0.692 |
|  | Within Groups | 62.136 | 2.589 |  |
|  | Total | 64.074 |  |  |
| 17 | Between Groups | 0.571 | 0.286 | 0.870 |
|  | Within Groups | 49.032 | 2.043 |  |
|  | Total | 49.603 |  |  |
| 18 | Between Groups | 3.585 | 1.793 | 0.530 |
|  | Within Groups | 66.052 | 2.752 |  |
|  | Total | 69.638 |  |  |
| 19 | Between Groups | 1.160 | 0.580 | 0.739 |
|  | Within Groups | 45.357 | 1.890 |  |
|  | Total | 46.517 |  |  |
| 20 | Between Groups | 1.282 | 0.641 | 0.770 |
|  | Within Groups | 58.090 | 2.420 |  |
|  | Total | 59.372 |  |  |
| 21 | Between Groups | 3.222 | 1.611 | 0.814 |
|  | Within Groups | 186.182 | 7.758 |  |
|  | Total | 189.404 |  |  |
| 22 | Between Groups | 8.435 | 4.217 | 0.607 |
|  | Within Groups | 198.261 | 8.261 |  |
|  | Total | 206.696 |  |  |
| 23 | Between Groups | 5.539 | 2.770 | 0.634 |
|  | Within Groups | 143.248 | 5.969 |  |
|  | Total | 148.788 |  |  |

| **Multiple Comparisons** | | | | | |
| --- | --- | --- | --- | --- | --- |
| Dependent Variable | Month | | Mean Difference (I-J) | Std. Error | Sig. |
| 1 | 1 | 3 | -0.40500 | 1.27994 | 0.754 |
|  |  | 6 | 0.80014 | 1.41042 | 0.576 |
|  | 3 | 1 | 0.40500 | 1.27994 | 0.754 |
|  |  | 6 | 1.20514 | 1.41042 | 0.401 |
|  | 6 | 1 | -0.80014 | 1.41042 | 0.576 |
|  |  | 3 | -1.20514 | 1.41042 | 0.401 |
| 2 | 1 | 3 | -0.30200 | 1.56769 | 0.849 |
|  |  | 6 | -2.19000 | 1.72751 | 0.217 |
|  | 3 | 1 | 0.30200 | 1.56769 | 0.849 |
|  |  | 6 | -1.88800 | 1.72751 | 0.285 |
|  | 6 | 1 | 2.19000 | 1.72751 | 0.217 |
|  |  | 3 | 1.88800 | 1.72751 | 0.285 |
| 3 | 1 | 3 | -0.89600 | 0.98382 | 0.371 |
|  |  | 6 | -0.49600 | 1.08412 | 0.651 |
|  | 3 | 1 | 0.89600 | 0.98382 | 0.371 |
|  |  | 6 | 0.40000 | 1.08412 | 0.715 |
|  | 6 | 1 | 0.49600 | 1.08412 | 0.651 |
|  |  | 3 | -0.40000 | 1.08412 | 0.715 |
| 4 | 1 | 3 | -0.43800 | 1.51258 | 0.775 |
|  |  | 6 | -0.68400 | 1.66678 | 0.685 |
|  | 3 | 1 | 0.43800 | 1.51258 | 0.775 |
|  |  | 6 | -0.24600 | 1.66678 | 0.884 |
|  | 6 | 1 | 0.68400 | 1.66678 | 0.685 |
|  |  | 3 | 0.24600 | 1.66678 | 0.884 |
| 5 | 1 | 3 | -0.61900 | 0.96511 | 0.527 |
|  |  | 6 | 0.01186 | 1.06350 | 0.991 |
|  | 3 | 1 | 0.61900 | 0.96511 | 0.527 |
|  |  | 6 | 0.63086 | 1.06350 | 0.559 |
|  | 6 | 1 | -0.01186 | 1.06350 | 0.991 |
|  |  | 3 | -0.63086 | 1.06350 | 0.559 |
| 6 | 1 | 3 | 1.02300 | 1.01769 | 0.325 |
|  |  | 6 | 0.59586 | 1.12144 | 0.600 |
|  | 3 | 1 | -1.02300 | 1.01769 | 0.325 |
|  |  | 6 | -0.42714 | 1.12144 | 0.707 |
|  | 6 | 1 | -0.59586 | 1.12144 | 0.600 |
|  |  | 3 | 0.42714 | 1.12144 | 0.707 |
| 7 | 1 | 3 | 0.53100 | 0.74784 | 0.485 |
|  |  | 6 | 0.32586 | 0.82408 | 0.696 |
|  | 3 | 1 | -0.53100 | 0.74784 | 0.485 |
|  |  | 6 | -0.20514 | 0.82408 | 0.806 |
|  | 6 | 1 | -0.32586 | 0.82408 | 0.696 |
|  |  | 3 | 0.20514 | 0.82408 | 0.806 |
| 8 | 1 | 3 | 0.04600 | 0.83243 | 0.956 |
|  |  | 6 | -1.47543 | 0.91729 | 0.121 |
|  | 3 | 1 | -0.04600 | 0.83243 | 0.956 |
|  |  | 6 | -1.52143 | 0.91729 | 0.110 |
|  | 6 | 1 | 1.47543 | 0.91729 | 0.121 |
|  |  | 3 | 1.52143 | 0.91729 | 0.110 |
| 9 | 1 | 3 | 1.16800 | 0.69917 | 0.108 |
|  |  | 6 | -0.03629 | 0.77044 | 0.963 |
|  | 3 | 1 | -1.16800 | 0.69917 | 0.108 |
|  |  | 6 | -1.20429 | 0.77044 | 0.131 |
|  | 6 | 1 | 0.03629 | 0.77044 | 0.963 |
|  |  | 3 | 1.20429 | 0.77044 | 0.131 |
| 10 | 1 | 3 | -0.57700 | 0.94622 | 0.548 |
|  |  | 6 | -0.43114 | 1.04269 | 0.683 |
|  | 3 | 1 | 0.57700 | 0.94622 | 0.548 |
|  |  | 6 | 0.14586 | 1.04269 | 0.890 |
|  | 6 | 1 | 0.43114 | 1.04269 | 0.683 |
|  |  | 3 | -0.14586 | 1.04269 | 0.890 |
| 11 | 1 | 3 | -0.08200 | 0.61484 | 0.895 |
|  |  | 6 | 0.09457 | 0.67752 | 0.890 |
|  | 3 | 1 | 0.08200 | 0.61484 | 0.895 |
|  |  | 6 | 0.17657 | 0.67752 | 0.797 |
|  | 6 | 1 | -0.09457 | 0.67752 | 0.890 |
|  |  | 3 | -0.17657 | 0.67752 | 0.797 |
| 12 | 1 | 3 | -1.24200 | 1.27522 | 0.340 |
|  |  | 6 | -0.59186 | 1.40522 | 0.677 |
|  | 3 | 1 | 1.24200 | 1.27522 | 0.340 |
|  |  | 6 | 0.65014 | 1.40522 | 0.648 |
|  | 6 | 1 | 0.59186 | 1.40522 | 0.677 |
|  |  | 3 | -0.65014 | 1.40522 | 0.648 |
| 13 | 1 | 3 | 0.62500 | 1.13788 | 0.588 |
|  |  | 6 | -0.06600 | 1.25388 | 0.958 |
|  | 3 | 1 | -0.62500 | 1.13788 | 0.588 |
|  |  | 6 | -0.69100 | 1.25388 | 0.587 |
|  | 6 | 1 | 0.06600 | 1.25388 | 0.958 |
|  |  | 3 | 0.69100 | 1.25388 | 0.587 |
| 14 | 1 | 3 | -0.30800 | 0.89533 | 0.734 |
|  |  | 6 | -0.11171 | 0.98660 | 0.911 |
|  | 3 | 1 | 0.30800 | 0.89533 | 0.734 |
|  |  | 6 | 0.19629 | 0.98660 | 0.844 |
|  | 6 | 1 | 0.11171 | 0.98660 | 0.911 |
|  |  | 3 | -0.19629 | 0.98660 | 0.844 |
| 15 | 1 | 3 | -0.01900 | 1.26199 | 0.988 |
|  |  | 6 | -0.70343 | 1.39065 | 0.618 |
|  | 3 | 1 | 0.01900 | 1.26199 | 0.988 |
|  |  | 6 | -0.68443 | 1.39065 | 0.627 |
|  | 6 | 1 | 0.70343 | 1.39065 | 0.618 |
|  |  | 3 | 0.68443 | 1.39065 | 0.627 |
| 16 | 1 | 3 | 0.24300 | 0.71958 | 0.739 |
|  |  | 6 | -0.44143 | 0.79294 | 0.583 |
|  | 3 | 1 | -0.24300 | 0.71958 | 0.739 |
|  |  | 6 | -0.68443 | 0.79294 | 0.397 |
|  | 6 | 1 | 0.44143 | 0.79294 | 0.583 |
|  |  | 3 | 0.68443 | 0.79294 | 0.397 |
| 17 | 1 | 3 | 0.20100 | 0.63922 | 0.756 |
|  |  | 6 | 0.36729 | 0.70438 | 0.607 |
|  | 3 | 1 | -0.20100 | 0.63922 | 0.756 |
|  |  | 6 | 0.16629 | 0.70438 | 0.815 |
|  | 6 | 1 | -0.36729 | 0.70438 | 0.607 |
|  |  | 3 | -0.16629 | 0.70438 | 0.815 |
| 18 | 1 | 3 | -0.42300 | 0.74191 | 0.574 |
|  |  | 6 | 0.50886 | 0.81755 | 0.540 |
|  | 3 | 1 | 0.42300 | 0.74191 | 0.574 |
|  |  | 6 | 0.93186 | 0.81755 | 0.266 |
|  | 6 | 1 | -0.50886 | 0.81755 | 0.540 |
|  |  | 3 | -0.93186 | 0.81755 | 0.266 |
| 19 | 1 | 3 | 0.45200 | 0.61480 | 0.469 |
|  |  | 6 | 0.38929 | 0.67747 | 0.571 |
|  | 3 | 1 | -0.45200 | 0.61480 | 0.469 |
|  |  | 6 | -0.06271 | 0.67747 | 0.927 |
|  | 6 | 1 | -0.38929 | 0.67747 | 0.571 |
|  |  | 3 | 0.06271 | 0.67747 | 0.927 |
| 20 | 1 | 3 | -0.44200 | 0.69576 | 0.531 |
|  |  | 6 | -0.46343 | 0.76669 | 0.551 |
|  | 3 | 1 | 0.44200 | 0.69576 | 0.531 |
|  |  | 6 | -0.02143 | 0.76669 | 0.978 |
|  | 6 | 1 | 0.46343 | 0.76669 | 0.551 |
|  |  | 3 | 0.02143 | 0.76669 | 0.978 |
| 21 | 1 | 3 | 0.02900 | 1.24560 | 0.982 |
|  |  | 6 | -0.77329 | 1.37258 | 0.578 |
|  | 3 | 1 | -0.02900 | 1.24560 | 0.982 |
|  |  | 6 | -0.80229 | 1.37258 | 0.564 |
|  | 6 | 1 | 0.77329 | 1.37258 | 0.578 |
|  |  | 3 | 0.80229 | 1.37258 | 0.564 |
| 22 | 1 | 3 | -0.10900 | 1.28537 | 0.933 |
|  |  | 6 | -1.32543 | 1.41641 | 0.359 |
|  | 3 | 1 | 0.10900 | 1.28537 | 0.933 |
|  |  | 6 | -1.21643 | 1.41641 | 0.399 |
|  | 6 | 1 | 1.32543 | 1.41641 | 0.359 |
|  |  | 3 | 1.21643 | 1.41641 | 0.399 |
| 23 | 1 | 3 | -0.79300 | 1.09258 | 0.475 |
|  |  | 6 | 0.28314 | 1.20397 | 0.816 |
|  | 3 | 1 | 0.79300 | 1.09258 | 0.475 |
|  |  | 6 | 1.07614 | 1.20397 | 0.380 |
|  | 6 | 1 | -0.28314 | 1.20397 | 0.816 |
|  |  | 3 | -1.07614 | 1.20397 | 0.380 |

**Figure S1.** Postoperative X-ray, CT and 3D model reconstruction images of Case 2.


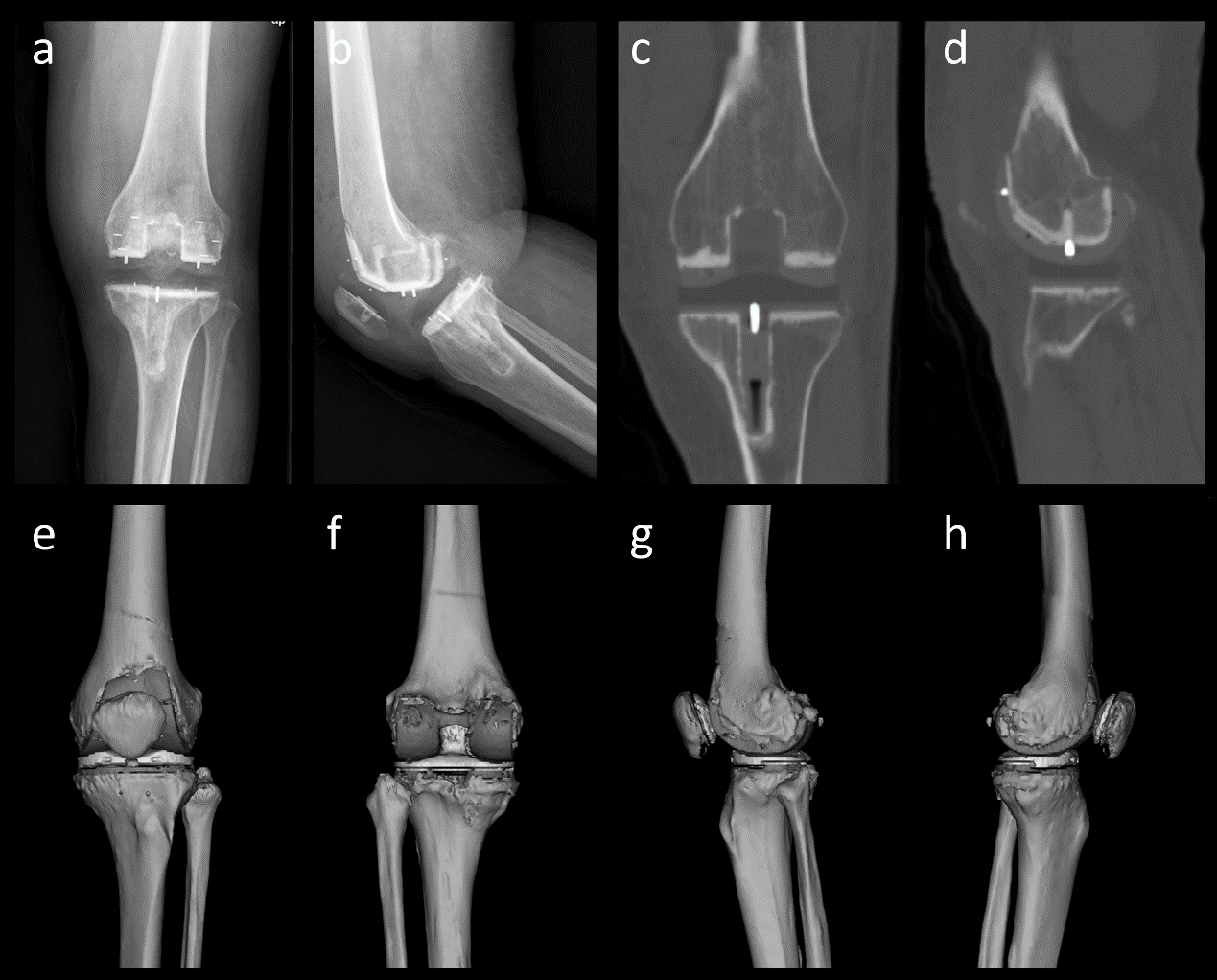


**Figure S2.** Postoperative X-ray, CT and 3D model reconstruction images of Case 3.


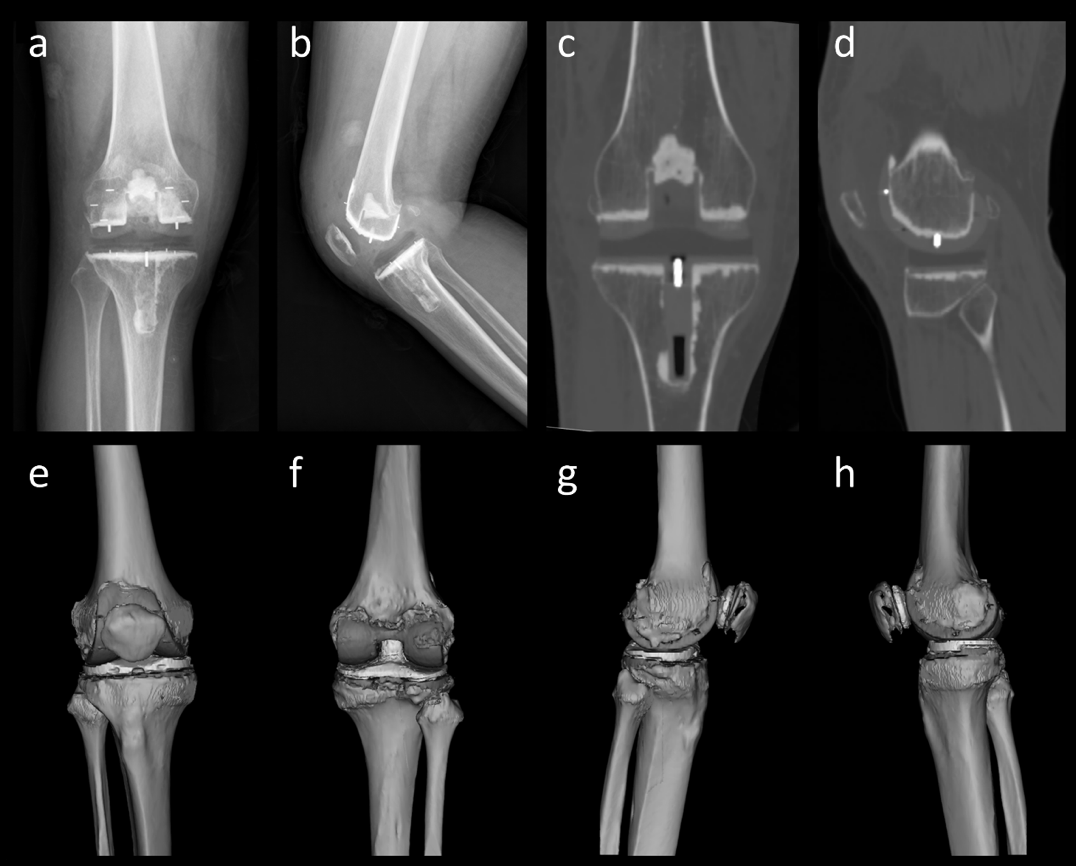


**Figure S3.** Postoperative X-ray, CT and 3D model reconstruction images of Case 4.


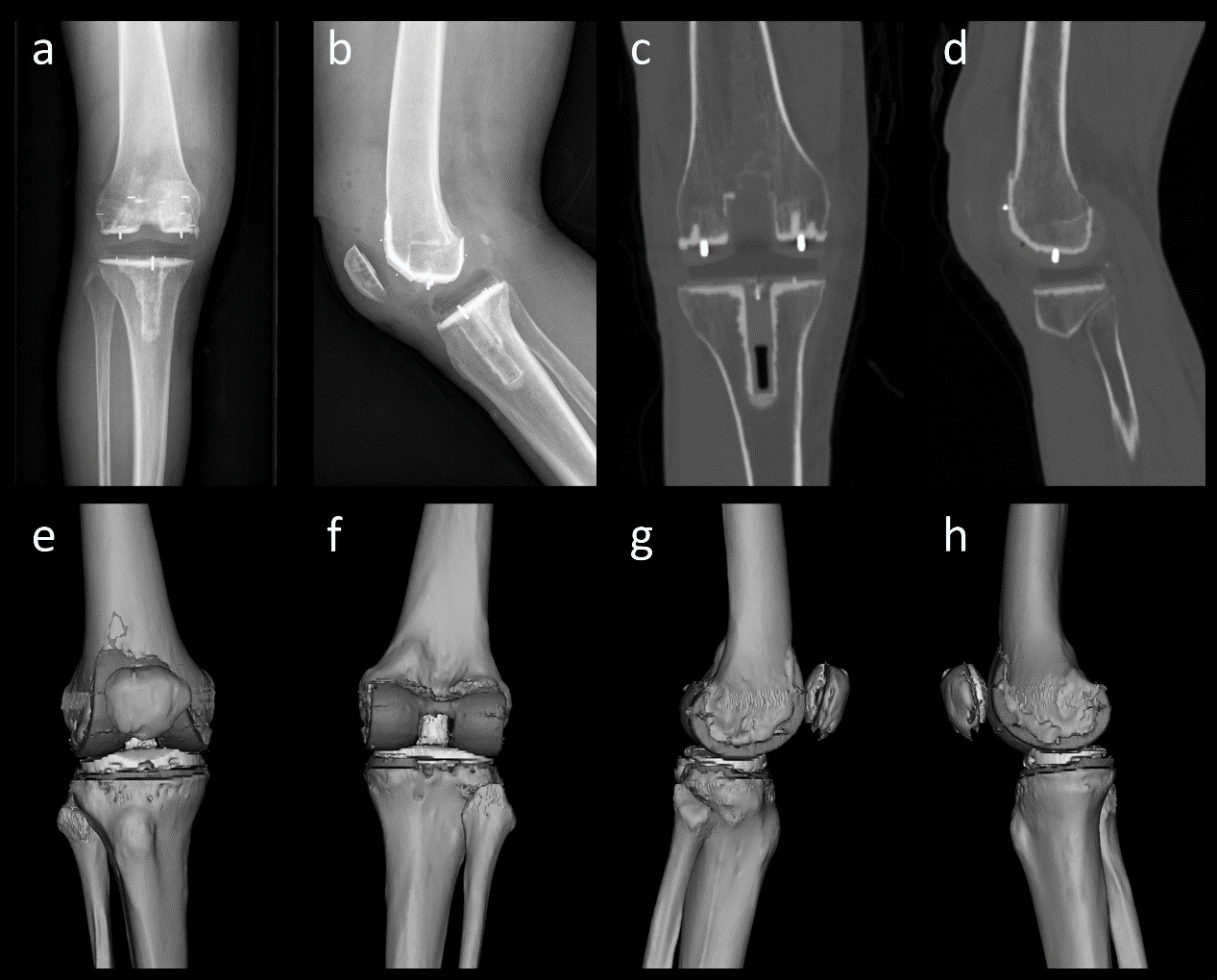


**Figure S4.** Postoperative X-ray, CT and 3D model reconstruction images of Case 5.


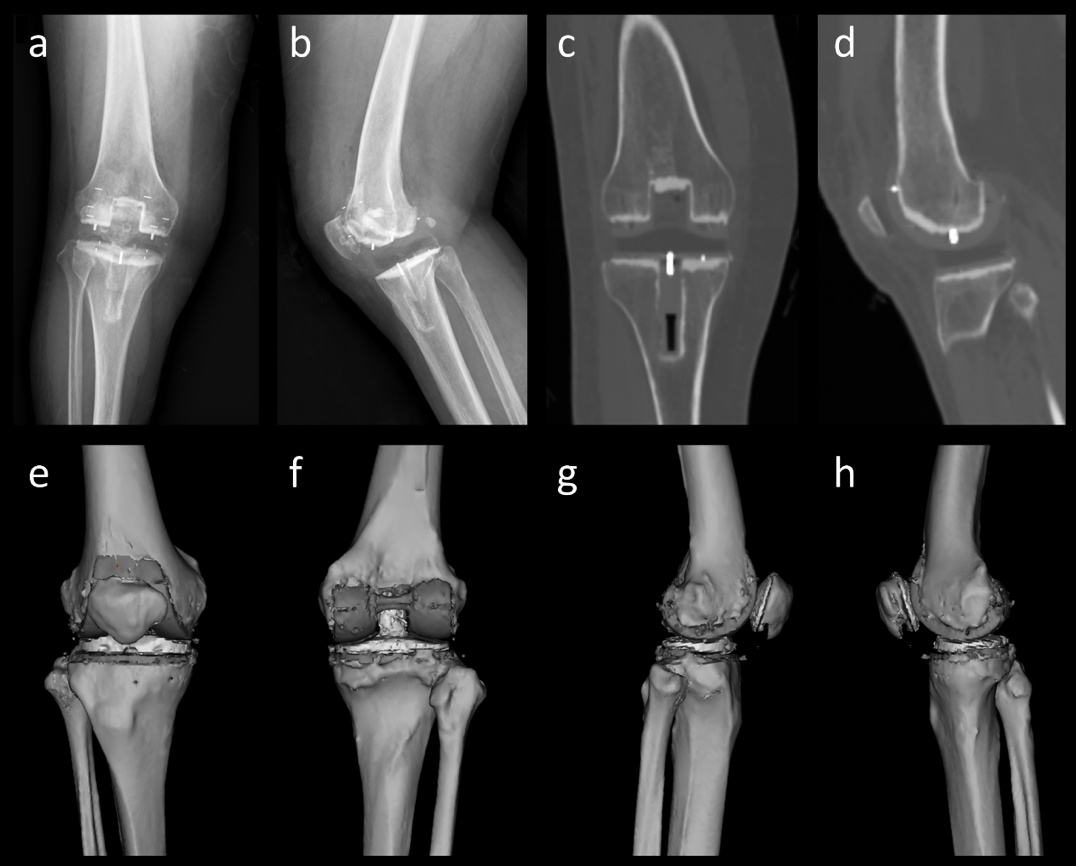


**Figure S5.** Postoperative X-ray, CT and 3D model reconstruction images of Case 6.


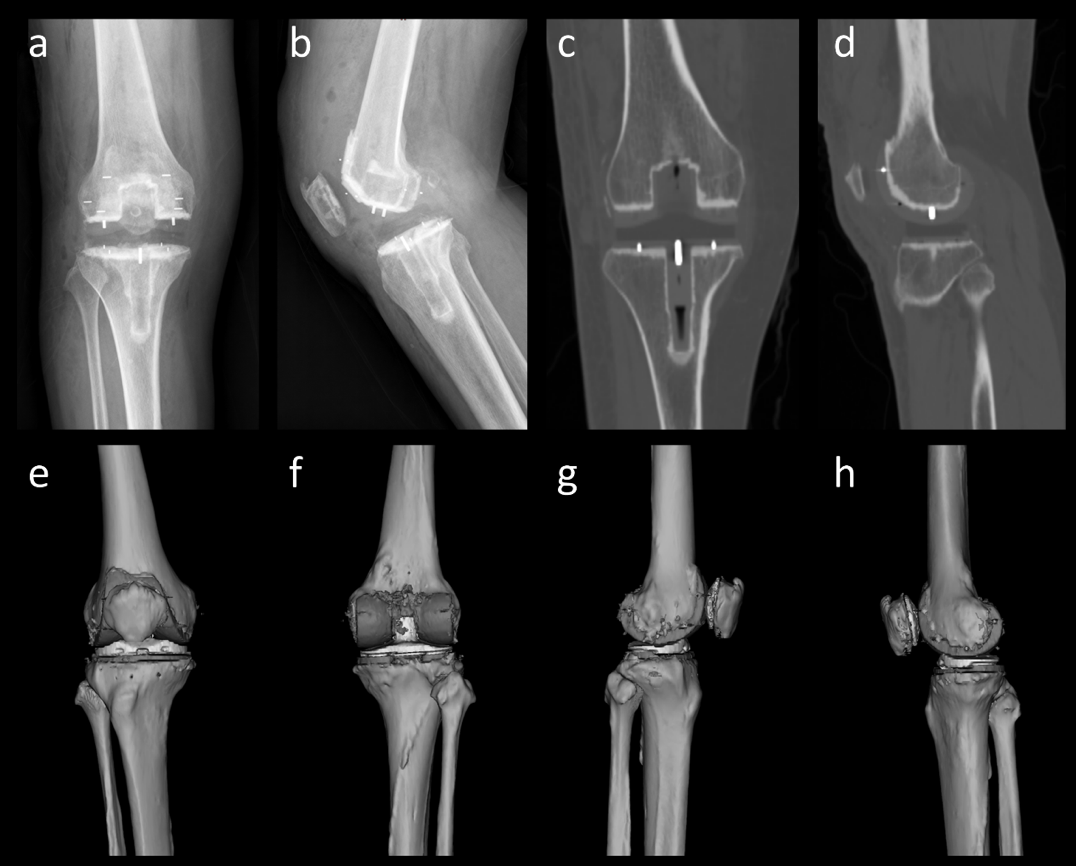


**Figure S6.** Postoperative X-ray, CT and 3D model reconstruction images of Case 7.


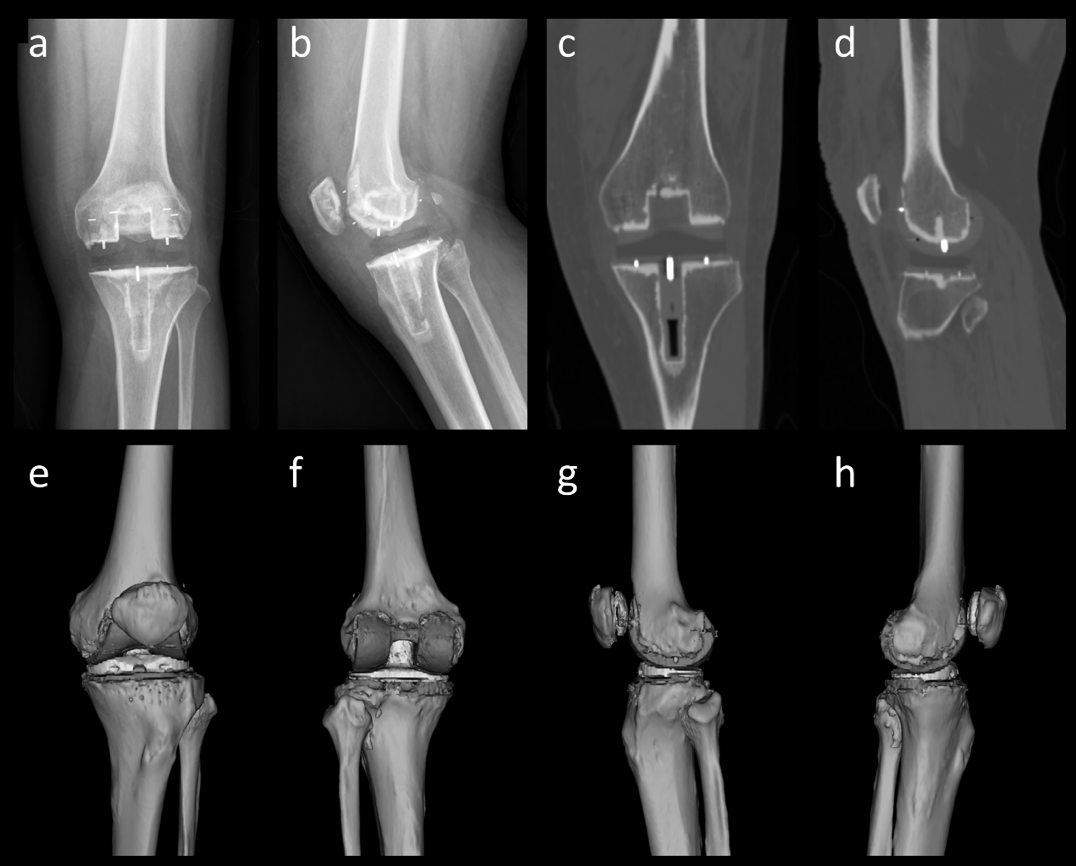


**Figure S7.** Postoperative X-ray, CT and 3D model reconstruction images of Case 8.


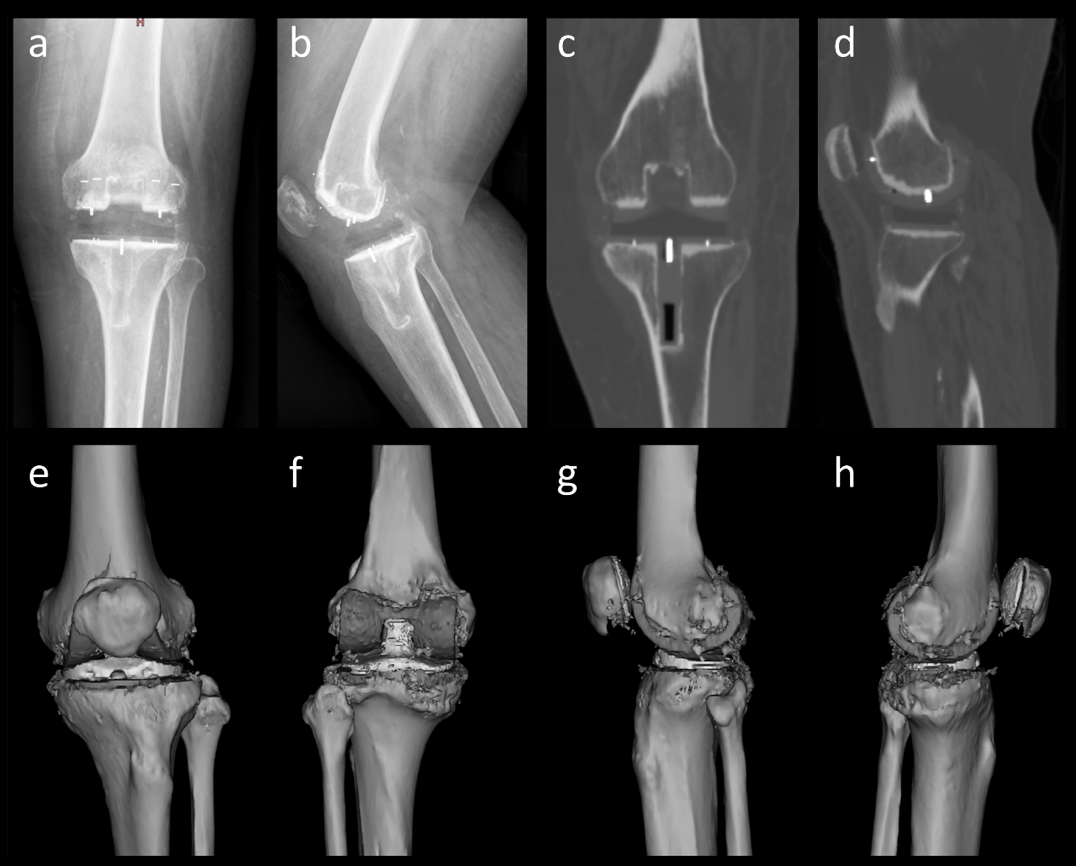


**Figure S8.** Postoperative X-ray, CT and 3D model reconstruction images of Case 9.


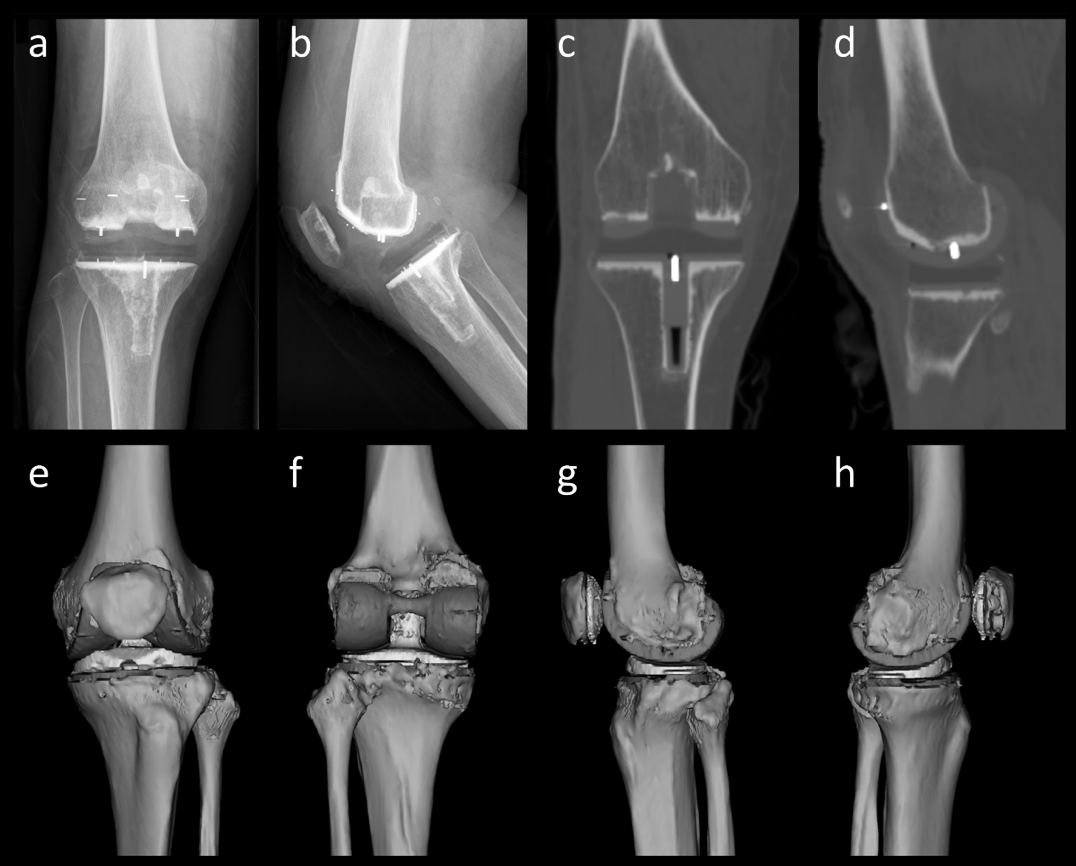


**Figure S9.** Postoperative X-ray, CT and 3D model reconstruction images of Case 10.


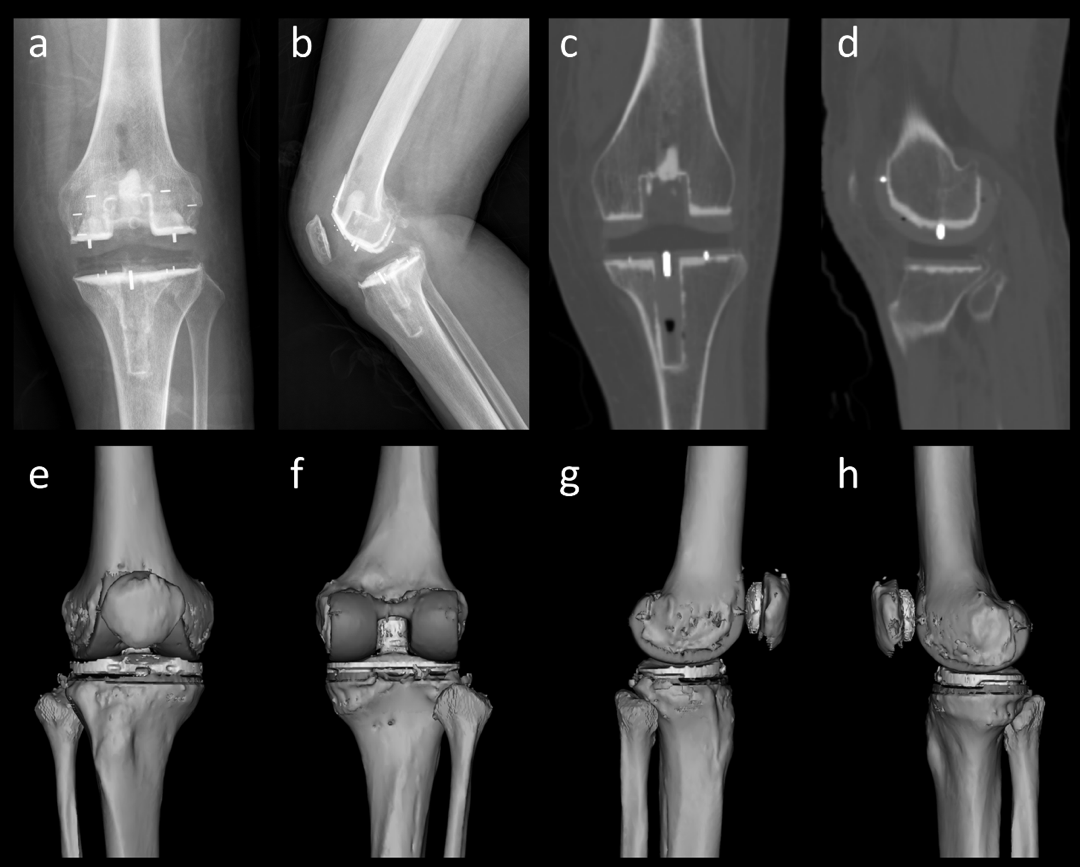


**Figure S1-Figure S9.** Postoperative X-ray frontal and lateral views of the other 9 patients (a,b), images of postoperative CT coronal intermediate and lateral condyle central layers (c,d), and a preview of the 3D reconstructed model (e-h) are shown. It can be seen that the joint prosthesis is positioned in the correct position (a,b), that the prosthesis is well integrated with the bone (c,d) and that the parts of the prosthesis are well aligned (e-h).


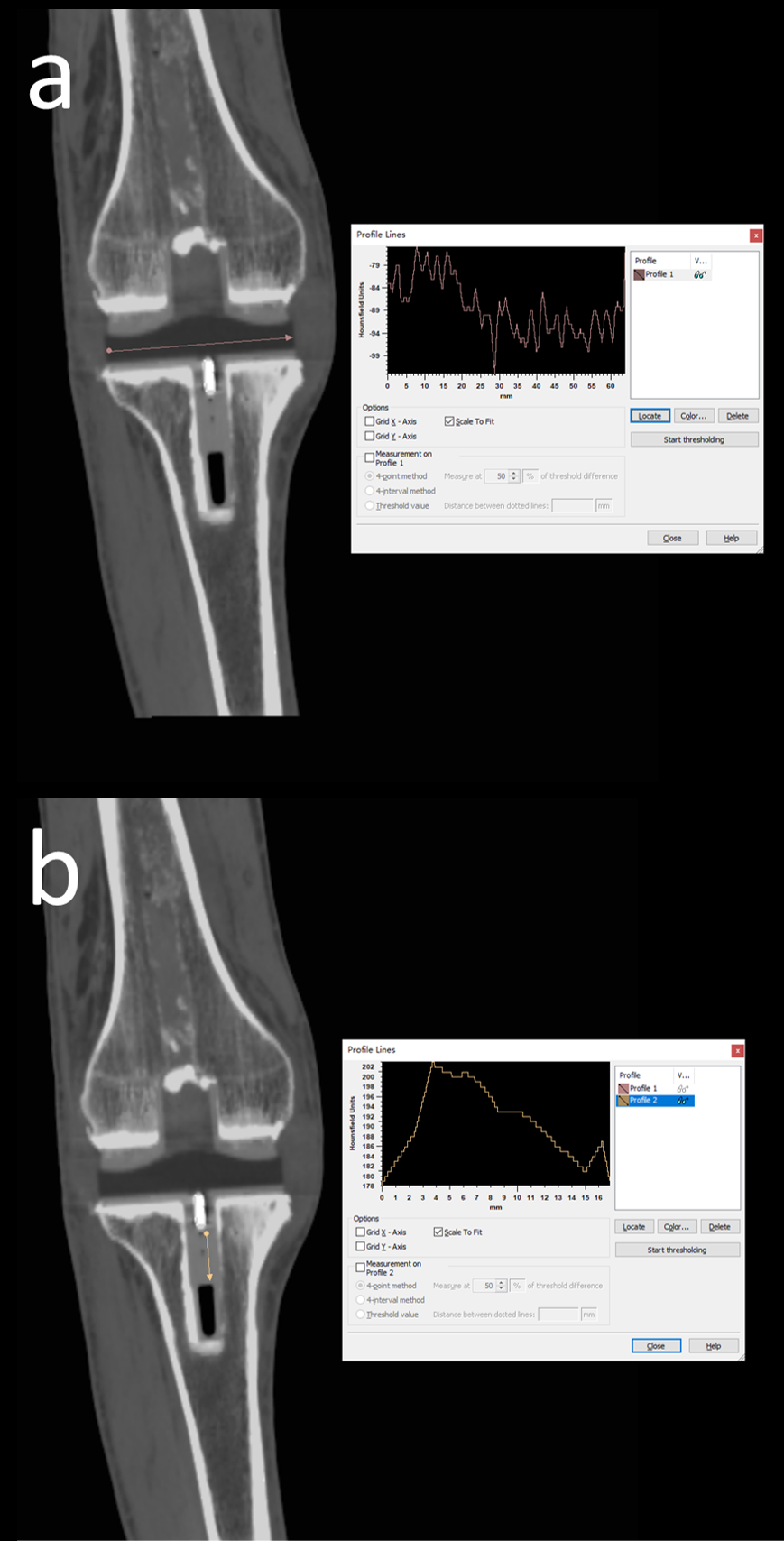


**Figure S10. PE spacer fluctuates between -100 and -75 on the transverse axis position.**

(a). The stem portion of the PEEK tibial prosthesis fluctuates between 178 and 202 on the longitudinal axis range. Such variability in the quantitative CT assessment is very small on CT. This result is also similar to the findings in table.2 and suggests that we can use these two components of the prosthesis as reference values for future quantitative CT assessments.


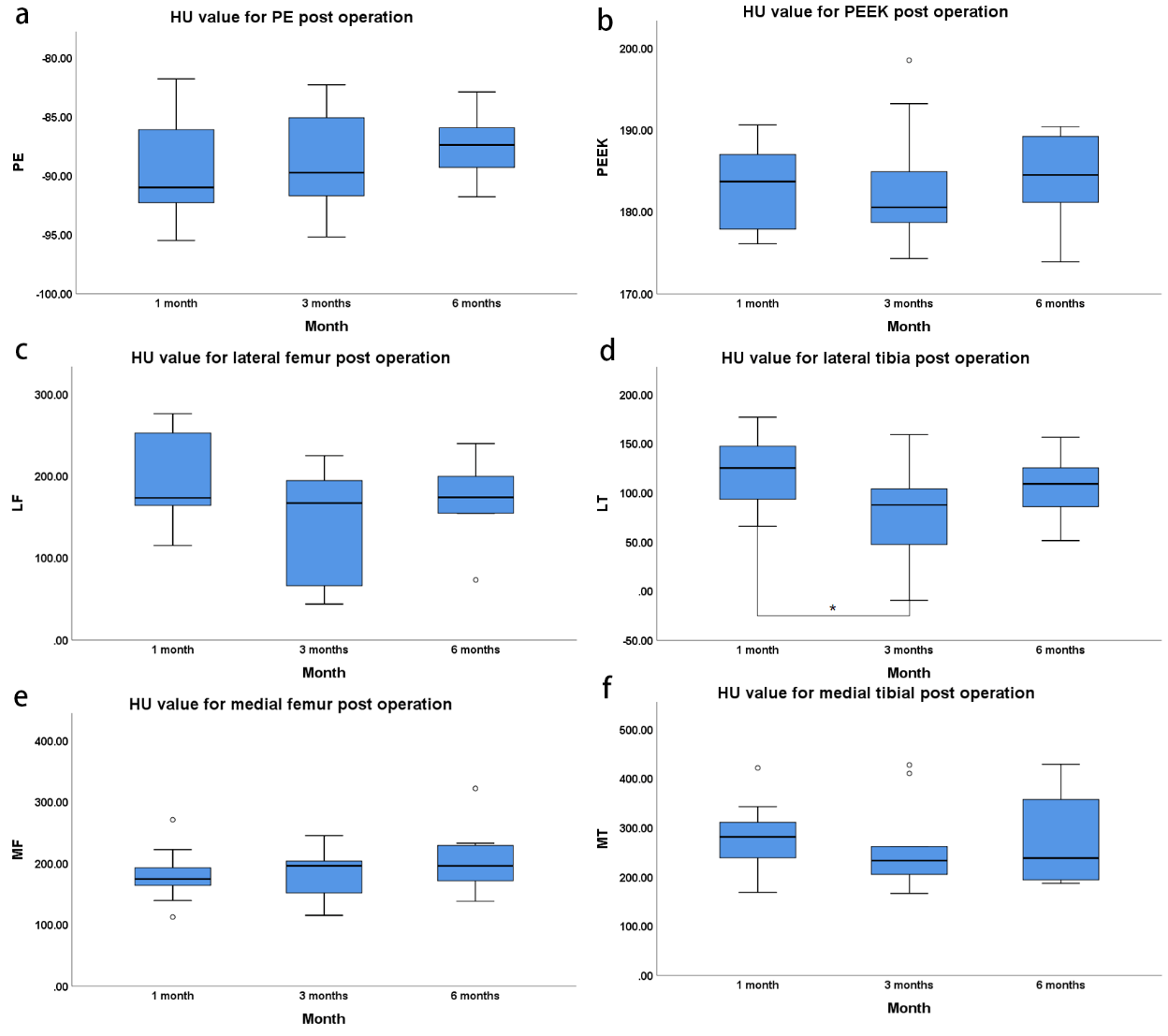


**Figure S11. Box plots of HU values at 1, 3 and 6 months for HXLPE, PEEK, lateral femur, lateral tibia, medial femur and medial tibial cancellous bone.**

According to the measurements, the values of PE and PEEK did not change significantly within 6 months (a,b) and can be used as a reference value for the measurements. The decrease in BMD of the lateral tibia was significant at 1-3 months (d), but otherwise there were no statistically significant changes in BMD (c-f). In terms of mean BMD, except for the medial femur, which increased from 1 to 6 months, BMD decreased at 3 months and increased at 6 months, but none of these increases were statistically significant, probably because our sample size was still pressed as insufficient.

1. Bonnin MP, de Kok A, Verstraete M, Van Hoof T, Van Der Straten C, Saffarini M, Victor J (2017) Popliteus impingement after TKA may occur with well-sized prostheses. Knee Surg Sports Traumatol Arthrosc 25:1720-1730. DOI 10.1007/s00167-016-4330-8

2. Du Z, Zhu Z, Yue B, Li Z, Wang Y (2018) Feasibility and Safety of a Cemented PEEK-on-PE Knee Replacement in a Goat Model: A Preliminary Study. Artif Organs 42:E204-E214. DOI 10.1111/aor.13101
